# Combined Effects of ATRA and ASA on HeLa Cell Viability, MAPK/AKT-Related Protein Abundance, and Intracellular VEGF: An Integrated In Vitro and In Silico Study

**DOI:** 10.3390/ph19101556

**Published:** 2026-10-01

**Authors:** Selim Afşar, Şeyma Dümür, Gazi Güner, Serkan Sarıkaya, Figen Efe Çamili, Gürhan Güney, Mine İslimye Taşkın, Dilek Düzgün Ergun, Öznur Dündar Akın, Çiğdem Usul Afşar, Abdurrahman Yiğit, Hafize Uzun

**Affiliations:** 1 Department of Obstetrics & Gynecology, Faculty of Medicine, Balıkesir University, 10020 Balıkesir, Turkey; drserkansarikaya@gmail.com (S.S.); efefigenefe@gmail.com (F.E.Ç.); guney.gurhan@mayo.edu (G.G.); minetaskin1302@yahoo.com.tr (M.İ.T.); 2 Department of Medical Biochemistry, Faculty of Medicine, Istanbul Atlas University, 34408 Istanbul, Turkey; seyma.dumur@atlas.edu.tr (Ş.D.); huzun59@hotmail.com (H.U.); 3 Department of Obstetrics & Gynecology, Cam & Sakura City Hospital, 34480 Istanbul, Turkey; dr.gaziguner01@gmail.com; 4 Department of Biophysics, Faculty of Medicine, Istanbul Aydın University, 34295 Istanbul, Turkey; dilekergun@aydin.edu.tr; 5 Department of Gynecology and Obstetrics, Istanbul Atlas University, 34408 Istanbul, Turkey; oznur.akin@atlas.edu.tr; 6 Department of Medical Oncology, Hamidiye Medical Faculty, Health Sciences University, 34668 Istanbul, Turkey; cigdemusul@yahoo.com; 7 Department of Medical Oncology, Mersin State Hospital, Health Sciences University, 33240 Mersin, Turkey; dryigit@yahoo.com.tr

**Keywords:** acetylsalicylic acid, all-trans retinoic acid, HeLa cells, MAPK/AKT-related protein abundance, intracellular VEGF, HIF-1α

## Abstract

**Background/Objectives**: All-trans retinoic acid (ATRA) and acetylsalicylic acid (ASA; aspirin) have been reported to influence cancer-associated signaling through distinct molecular mechanisms, but their combined effects on cervical cancer cells remain incompletely characterized. This study evaluated the individual and combined effects of ATRA and ASA on HeLa human cervical cancer cells and used computational analyses as supportive, hypothesis-generating mechanistic contextualization. **Methods**: Cell viability was assessed by MTT assay after treatment with increasing concentrations of ATRA and ASA for 24, 48, and 72 h. The abundance of total ERK1/2, phospho-AKT (Ser473), total p38 MAPK, and total JNK was assessed by Western blotting, while intracellular HIF-1α and VEGF concentrations were measured in cell lysates by ELISA. Computational analyses included cross-validated response modeling, bootstrap estimation of Bliss excess inhibition, STRING network topology and enrichment, SwissTargetPrediction/SEA consensus target prediction, TCGA-CESC survival analysis, and redocking-validated molecular docking. **Results**: ATRA and ASA reduced HeLa cell viability in a dose- and time-dependent manner. Treatment was associated with lower total ERK1/2 abundance and lower phospho-AKT (Ser473) abundance, whereas ASA-containing conditions were associated with higher stress-associated MAPK markers, particularly JNK. Intracellular VEGF concentrations were lower after ATRA, ASA, and combined treatment, while intracellular HIF-1α concentrations were not significantly altered under normoxic culture conditions. Computational response modeling showed high predictive accuracy for ATRA and ASA, while bootstrap Bliss analysis indicated dose-dependent interaction behavior rather than uniform synergy. STRING analysis supported pathway-level coherence among retinoid, COX/prostaglandin, MAPK/AKT, hypoxia, and angiogenesis-related modules. TCGA-CESC analyses associated VEGFA, HIF1A, and PTGS2 with adverse survival outcomes in adjusted models. Docking supported ATRA–RARG and salicylate–COX-2 pose plausibility but did not support direct VEGFR2 or AKT1 targeting by ASA or salicylate. **Conclusions**: These findings suggest that ATRA and ASA reduce HeLa cell viability and are associated with altered MAPK/AKT-related protein abundance and lower intracellular VEGF concentrations in HeLa cervical cancer cells. The computational results provide pathway-level support and target prioritization for future validation but should not be interpreted as proof of direct binding, causal pathway mediation, treatment efficacy, or clinical benefit.

## 1. Introduction

Cervical cancer remains a major global health burden and one of the most common malignancies affecting women worldwide [1]. Persistent high-risk human papillomavirus infection, particularly HPV16 and HPV18, is the central etiological driver, but malignant progression also depends on host–cell signaling networks that regulate proliferation, survival, metabolic adaptation, inflammation, and angiogenesis. Although vaccination, screening, surgery, radiotherapy, chemotherapy, and immunotherapy have improved prevention and treatment, advanced, recurrent, and therapy-resistant disease still requires additional molecularly informed strategies. Recent reviews have highlighted HPV-associated kinase signaling, including PI3K/AKT/mTOR and MAPK-related pathways, as important contributors to cervical cancer biology and drug resistance [2,3]. HeLa cells, derived from HPV18-positive cervical adenocarcinoma, remain a widely used model for studying cervical cancer viability, survival signaling, hypoxia-related responses, and treatment-associated molecular changes. They therefore provide a relevant in vitro system for testing whether pharmacologically accessible agents can modulate survival- and angiogenesis-related signaling in cervical cancer.

All-trans retinoic acid (ATRA), the biologically active derivative of vitamin A, regulates cell differentiation, proliferation, apoptosis-related transcriptional programs, and developmental patterning through retinoic acid receptors (RARs) and retinoid X receptors (RXRs). ATRA has produced major clinical success in acute promyelocytic leukemia, but its activity in solid tumors has been more variable, highlighting the context-dependent nature of retinoid signaling [4,5,6]. Retinoid signaling may also influence cervical carcinogenesis and the progression of cervical intraepithelial neoplasia [7,8]. Mechanistically, ATRA can act through genomic receptor-mediated transcription as well as non-genomic crosstalk with kinase cascades, including MAPK-related signaling [4,5,6,9]. MAPKs include ERK1/2, c-Jun N-terminal kinase (JNK), and p38 MAPK; ERK1/2 is generally associated with proliferation and survival signaling, whereas JNK and p38 MAPK are commonly linked to cellular stress responses, autophagy, and apoptosis-related signaling [10,11]. These properties make ATRA a biologically plausible candidate for evaluating signaling modulation in cervical cancer cells, while requiring cautious interpretation because retinoid responses differ substantially across tumor types and experimental contexts.

Chronic inflammation is closely connected to cancer initiation and progression, partly through cyclooxygenase-2 (COX-2)-dependent prostaglandin E2 (PGE2) signaling. COX-2/PGE2 activity can support tumor-cell proliferation, migration, apoptosis resistance, epithelial–mesenchymal transition, immune escape, VEGF-mediated angiogenesis, and therapeutic resistance [12,13]. Aspirin, also known as acetylsalicylic acid (ASA), is a non-steroidal anti-inflammatory drug that inhibits COX-1 and COX-2, but its anticancer-relevant effects may also involve COX-independent pathways, including modulation of NF-κB, Wnt/β-catenin, cytokine signaling, autophagy, DNA repair, and cell-survival networks [14,15]. A Pharmaceuticals review specifically highlighted the pharmacological complexity of aspirin as an anticancer agent and emphasized that low-dose clinical effects are strongly linked to platelet COX-1 inhibition, whereas extrapolation of direct tumor-cell effects from in vitro models requires caution [14]. In cervical cancer models, ASA has been reported to promote apoptosis-related responses by inhibiting ERK1/2 signaling and increasing caspase activity in HeLa cells [16], and to inhibit ErbB2-associated survival signaling in cervical cancer cells [17]. These observations support evaluating ASA not only as an anti-inflammatory compound but also as a modulator of cervical cancer survival and angiogenesis-related signaling.

The MAPK/ERK and PI3K/AKT pathways are central survival networks that regulate proliferation, apoptosis resistance, metabolism, motility, and treatment response in cancer [3,11]. In HPV-positive cervical cancer cells, PI3K/AKT/mTOR signaling contributes to virus–host-cell crosstalk under both normoxic and hypoxic conditions, and hypoxia-related pathway changes can influence therapeutic responses [3]. ERK and AKT signaling have also been linked to angiogenic output through regulation of HIF-1α and vascular endothelial growth factor (VEGF), although VEGF regulation is context-dependent and may involve both HIF-1α-dependent and HIF-1α-independent mechanisms [18,19,20]. The adaptation of tumor cells to low oxygen tension within the tumor microenvironment depends in part on HIF-1α-mediated transcriptional programs, which promote angiogenesis and support metastatic potential through VEGF induction [18,21]. In HeLa cells, ROS-induced ERK and HIF-1α activation has been reported to contribute to survival-related cellular adaptations under serum-deprived normoxic conditions [22]. Together, these findings justify integrated evaluation of ERK1/2, phospho-AKT, stress-associated MAPKs, HIF-1α, and VEGF when assessing the molecular effects of ATRA and ASA in cervical cancer cells.

The combination of ATRA and ASA is biologically plausible because the two agents may influence complementary components of cancer-associated signaling. ATRA primarily acts through retinoid receptor-mediated transcription and context-dependent crosstalk with MAPK-related pathways, whereas ASA can modulate COX/prostaglandin biology, inflammation-associated mediators, platelet-related mechanisms, and selected tumor-cell survival pathways [4,5,6,9,12,13,14,15]. However, the combined effects of ATRA and ASA have not been systematically investigated in HeLa human cervical cancer cells with respect to MAPK/AKT-related protein abundance and the HIF-1α/VEGF angiogenesis-related axis. Moreover, few studies have integrated in vitro ATRA + ASA treatment with computational target prediction, protein-network analysis, patient-cohort survival analysis, and docking-based structural assessment. Based on this gap, we hypothesized that combined ATRA and ASA treatment would exert complementary anti-proliferative and signaling-modulatory effects by reducing HeLa cell viability, altering total ERK1/2, phospho-AKT, total p38 MAPK, and total JNK abundance, and decreasing intracellular VEGF concentrations without necessarily producing parallel modulation of intracellular HIF-1α. Therefore, this study evaluated the individual and combined effects of ATRA and ASA on HeLa cell viability and on total ERK1/2, phospho-AKT, total p38 MAPK, total JNK, HIF-1α, and VEGF measurements in an in vitro cervical cancer model, with computational analyses used as supporting mechanistic context rather than confirmatory evidence of direct target engagement or clinical efficacy.

## 2. Results

### 2.1. In Vitro Effects of ATRA/ASA on HeLa Cells by MTT Analysis at 570 nm

The anti-proliferative activity of ATRA and ASA was evaluated in HeLa cells using the MTT assay. Cells were treated with increasing concentrations of ATRA (2.5–100 μM) or ASA (0.25–10 mM) for 24, 48, and 72 h, and IC_50_ values were calculated for each treatment duration. Figure 1 presents the single-agent dose- and time–response curves for ATRA and ASA. Both agents reduced HeLa cell viability in a dose- and time-dependent manner, with lower IC_50_ values at longer exposure times; the detailed IC_50_ estimates and statistical comparisons are reported below.

Across all MTT measurements, both agents produced significant dose- and time-dependent reductions in HeLa cell viability, with significant dose × time interactions for both ATRA and ASA. The fitted ATRA IC_50_ decreased from 111.72 µM at 24 h to 27.21 µM at 48 h and 13.28 µM at 72 h. All tested ATRA concentrations differed significantly from their matched controls after Holm correction. ASA showed the same temporal decrease in fitted IC_50_ estimates. The fitted ASA IC_50_ decreased from 13.02 mM at 24 h to 2.85 mM at 48 h and 2.41 mM at 72 h. IC_50_ values represent fitted estimates derived from the tested concentration ranges under the MTT assay conditions and should not be interpreted as absolute potency values outside these experimental settings. Holm-corrected comparisons were significant at all tested ASA conditions except 0.25 mM at 24 h (adjusted *p* = 0.084). Together, these findings demonstrate progressively greater reduction in cell viability with increasing concentration and exposure duration (Figure 1). At 48 h, ATRA, ASA, and ATRA + ASA each reduced HeLa cell viability relative to control, with the combined treatment showing the lowest mean viability (Figure 2).

At 48 h, exploratory fixed-ratio interaction analysis suggested dose-dependent differences in the combined ATRA + ASA response. The lowest fixed-ratio pair (2.5 µM ATRA + 0.25 mM ASA) yielded 86.6% viability compared with a Bliss expectation of 89.1%, corresponding to a small excess inhibition of +2.6 percentage points. This pair was classified as synergistic by Chou–Talalay and Loewe criteria but remained within the prespecified Bliss additive interval. The 5 µM + 0.5 mM pair was additive across all three frameworks, whereas 25 µM + 2.5 mM showed consistent antagonism and the highest-dose comparisons produced divergent estimates near extreme effects. Because matched combination-control conditions and a full dose–response matrix were not available, these findings should be interpreted as exploratory interaction estimates rather than definitive pharmacological synergy modeling (Figure 3). Complete numerical fixed-ratio interaction metrics, including Bliss excess inhibition, bootstrap confidence intervals, Chou–Talalay combination index, and Loewe additivity values, are provided in Supplementary Table S1.

### 2.2. Western Blot Analysis Results

Western blot analysis was performed to determine whether ATRA, ASA, and their combination altered selected MAPK/AKT-related protein measurements in HeLa cells. Band intensities were normalized to β-actin and expressed relative to the control group. Total ERK1/2 abundance was reduced in all treatment groups, with the greatest reduction after combined ATRA + ASA treatment. Phospho-AKT (Ser473) abundance was also lowest in the combined-treatment group. In contrast, total p38 MAPK and total JNK followed a stress-associated pattern: p38 MAPK increased numerically mainly in ASA-containing groups, whereas JNK increased significantly after ASA and ATRA + ASA treatment. Because paired phosphorylated and total measurements were not available for all pathway components, these data should be interpreted as treatment-associated changes in selected protein abundance rather than definitive evidence of global MAPK/AKT pathway activation or inhibition (Figure 4).

One-way ANOVA showed significant treatment effects for total ERK1/2, phospho-AKT (Ser473), total p38 MAPK, and total JNK. Relative to Control (1.00), total ERK1/2 abundance was reduced to 0.60 ± 0.30 with ATRA, 0.57 ± 0.28 with ASA, and 0.21 ± 0.08 with ATRA + ASA; all three Holm-corrected comparisons were significant. Phospho-AKT (Ser473) abundance was lowest with ATRA + ASA (0.38 ± 0.13; adjusted *p* = 0.0182), whereas the single-agent comparisons did not remain significant after correction.

p38 MAPK increased numerically with ASA (1.56 ± 0.41) and ATRA + ASA (1.48 ± 0.57), but neither comparison with Control was significant after Holm correction. JNK increased to 2.72 ± 1.08 with ASA and 2.03 ± 0.77 with ATRA + ASA, and both increases remained significant. Thus, the treatments were associated with lower ERK1/2 and phospho-AKT abundance, while ASA-containing conditions favored higher stress-associated MAPK markers. These findings indicate altered survival- and stress-associated signaling measurements but do not establish the directionality, sequence, or functional activity of the broader MAPK/AKT pathways (Figure 4).

### 2.3. ELISA Analysis of HIF-1α and VEGF

ELISA analysis showed that intracellular VEGF concentrations measured in HeLa cell lysates were significantly lower after treatment with ATRA, ASA, and ATRA + ASA, with the combined treatment showing the lowest measured VEGF concentration among the tested groups (Figure 5). In contrast, intracellular HIF-1α concentrations did not differ significantly among the control and treatment groups under standard normoxic culture conditions. Because VEGF was measured in cell lysates rather than conditioned medium and was not independently normalized to total protein content or cell number after treatment, these findings describe lower measured intracellular VEGF concentrations but do not establish reduced VEGF secretion or specific anti-angiogenic activity.

HIF-1α declined from 1.78 ± 0.78 ng/mL in Control to 1.08 ± 0.16, 1.29 ± 0.67, and 0.98 ± 0.16 ng/mL after ATRA, ASA, and ATRA + ASA treatment, respectively, but the overall treatment effect was not significant and no Holm-corrected comparison with Control reached significance. VEGF decreased from 447.54 ± 122.43 pg/mL in Control to 232.61 ± 44.50 pg/mL with ATRA, 220.81 ± 43.01 pg/mL with ASA, and 173.03 ± 38.26 pg/mL with ATRA + ASA. All VEGF reductions remained significant after Holm correction (adjusted *p* = 0.0096, 0.0096, and 0.0059, respectively; Figure 5).

To determine whether the combined ATRA + ASA response differed from the pattern expected from the individual agents, observed combined-treatment values were compared with expected independent-effect responses calculated from normalized ATRA-only and ASA-only values (Figure 6). Total ERK1/2, phospho-AKT (Ser473), total p38 MAPK, total JNK, and HIF-1α were broadly consistent with their expected independent-effect responses, suggesting that these endpoints were largely explained by the individual effects of ATRA and/or ASA. VEGF was the exception, showing a significant ATRA × ASA interaction (*p* = 0.0094) and identifying intracellular VEGF concentration as the principal interaction-dependent measured endpoint.

Two-factor factorial ANOVA further separated the main effects of ATRA and ASA from their interaction. Total ERK1/2 and phospho-AKT (Ser473) showed significant main effects of both ATRA and ASA without significant interaction terms, consistent with treatment-associated reductions in selected survival-related protein measurements. Total p38 MAPK and total JNK were primarily ASA-driven, whereas HIF-1α showed only a modest ATRA main effect and no significant interaction. VEGF showed significant ATRA and ASA main effects together with the only significant ATRA × ASA interaction (*p* = 0.0094). The observed combined-treatment intracellular VEGF concentration was higher than predicted by the multiplicative independent-effect expectation, indicating that the combined response differed from the independent-effect model but should not be interpreted as synergistic VEGF suppression or as evidence of reduced VEGF secretion (Figure 6).

### 2.4. Computational Target Prediction, Survival Analysis, and Molecular Docking Results

Integrated computational analysis was performed to assess response modeling, pathway-level coherence, target plausibility, clinical relevance, and structural support. Cross-validated response surfaces reproduced the dose- and time-dependent viability patterns with high predictive accuracy for ATRA (cross-validated R^2^ = 0.992; RMSE = 2.57 percentage points) and ASA (cross-validated R^2^ = 0.964; RMSE = 4.59 points; Figure 7A). Bootstrap Bliss analysis showed that the 2.5 µM ATRA plus 0.25 mM ASA pair produced a small positive deviation (+2.58 points, 95% CI 0.36–4.64) that remained below the prespecified synergy threshold, whereas the 25 µM plus 2.5 mM pair showed antagonism (−9.74 points, 95% CI −11.63 to −7.37; Figure 7B). Thus, the computational interaction analysis supports dose-dependent combination behavior rather than uniform synergy, while the descriptive biomarker profile is summarized in Figure 7C.

The 14-protein STRING network comprised 62 associations, corresponding to a density of 0.681 (Figure 8A). AKT1 ranked first and connected to all other proteins (degree = 13), followed by MAPK1 and MAPK3 (degree = 12 each), MAPK14 (degree = 11), and TP53 (degree = 10; Figure 8B). Enrichment of the 13 correctly mapped proteins supported cancer pathways, VEGF signaling, oxidative-stress response, angiogenesis, MAPK signaling, retinoic-acid receptor signaling, HIF-1 signaling, and PI3K–AKT signaling after false-discovery-rate correction (Figure 8C; Supplementary Figure S1). These findings support coherence among prespecified biological modules rather than unbiased pathway discovery.

SwissTargetPrediction and SEA identified 15 consensus targets for ATRA, 31 for ASA, and 38 for salicylate (Figure 9). The ATRA consensus was enriched for retinoid receptors, including RARG, RARA, RARB, RXRA, RXRB, and RXRG, whereas ASA and salicylate shared PTGS1, PTGS2, carbonic anhydrases, and several metabolic or signaling-related targets. The larger salicylate consensus indicates broader predicted target coverage but should not be interpreted as potency or efficacy.

In TCGA-CESC, higher VEGFA expression was associated with poorer overall survival by median-split Kaplan–Meier analysis (log-rank *p* = 0.0020) and adjusted continuous-expression Cox regression (HR per SD = 1.417, 95% CI 1.092–1.838; FDR = 0.0232; Figure 10B). HIF1A was not significant by median split (*p* = 0.259) but was significant in the adjusted continuous model (HR = 1.503, 95% CI 1.154–1.958; FDR = 0.0099), indicating sensitivity to expression modeling and distinguishing patient-cohort transcriptomic associations from the unchanged HIF-1α protein levels observed in HeLa cell lysates. PTGS2 was associated with poorer overall survival (HR = 1.481, 95% CI 1.163–1.885; FDR = 0.0099) and progression-free interval (HR = 1.415, 95% CI 1.120–1.787; FDR = 0.0286), although the progression-free interval proportional-hazards test was significant (*p* = 0.043) and warrants caution. JUN and TP53 were also FDR-significant for overall survival, with HRs of 1.298 and 0.796, respectively. The integrated computational summary is presented in Figure 10.

Native-ligand redocking reproduced reference poses for RARG, COX-2, VEGFR2, and AKT1, with best-pose RMSDs ranging from 0.257 to 0.686 Å; all satisfied the prespecified ≤2.0 Å criterion (Figure 10C). ATRA reproduced the RARG native-ligand score closely (−11.244 and −11.536 kcal/mol for neutral and anionic states, respectively, versus −11.372 kcal/mol for native REA). Salicylate similarly reproduced the noncovalent COX-2 native-ligand score (−6.087 and −6.177 versus −6.171 kcal/mol for native SAL). ASA yielded −6.820 and −6.822 kcal/mol at COX-2, but these poses do not represent Ser530 acetylation. At VEGFR2, ASA and salicylate scored −5.638 to −6.039 kcal/mol compared with −12.231 kcal/mol for native BAX; at AKT1, study ligands scored −5.840 to −6.269 kcal/mol compared with −9.658 kcal/mol for native 0XZ. These within-system differences provide weak support for direct VEGFR2 or AKT1 binding and argue against presenting either protein as a validated direct target of ASA or salicylate (Figure 10D).

## 3. Discussion

This study shows that ATRA and ASA reduce HeLa cell viability in a dose- and time-dependent manner and alter selected MAPK/AKT-related protein measurements in vitro. Treatment-associated reductions were observed for total ERK1/2, phospho-AKT (Ser473), and intracellular VEGF concentrations, particularly after combined ATRA + ASA treatment. In contrast, total p38 MAPK and total JNK did not follow the same reduction pattern and were mainly influenced by ASA-containing treatments. VEGF was the only measured marker showing a significant ATRA × ASA interaction, while intracellular HIF-1α concentrations were not significantly altered under normoxic culture conditions. Together, these findings indicate that ATRA and ASA are associated with lower survival-related protein measurements and lower intracellular VEGF concentrations in HeLa cells. However, because paired phospho/total analyses were incomplete, secreted VEGF was not measured, and HIF-1α stabilization controls were not included, these data should not be interpreted as proof of global MAPK/AKT pathway inhibition, reduced VEGF secretion, or an HIF-1α-independent regulatory mechanism.

Results from this study indicate that ATRA and ASA reduce HeLa cell viability while concurrently lowering total ERK1/2 and phospho-AKT (Ser473) abundance in vitro. The MAPK/ERK and PI3K/AKT pathways are intracellular signaling cascades involved in cell growth, proliferation, survival, apoptosis, motility, and invasion. Dual ERK and AKT inhibitors have potential clinical applications in cancer therapy [11]. In this context, the present findings suggest that ATRA and ASA are associated with lower selected survival-related protein measurements in HeLa cells, although additional pathway-level analyses would be required to confirm broader inhibition of the MAPK/ERK and PI3K/AKT pathways.

Previous studies have reported that ATRA can reduce VEGF expression or angiogenesis-related output in human keratinocytes and hematologic cancer models [19,20]. Evidence suggests that ATRA may regulate VEGF and related angiogenesis pathways in a context-dependent manner, thereby contributing to reduced tumor growth, angiogenesis, and metastasis in selected experimental systems [19,20]. Consistent with these reports, the present study found lower intracellular VEGF concentrations in HeLa cell lysates after ATRA treatment. Regulation of VEGF expression involves multiple factors, with hypoxia identified as a predominant inducer. HIF-1α is widely recognized as a principal mediator of angiogenesis, particularly in solid tumors [18]. To clarify whether lower VEGF measurements were accompanied by detectable HIF-1α modulation, we examined intracellular HIF-1α concentrations in HeLa cell lysates. HIF-1α concentrations were not significantly altered by ATRA treatment under the normoxic conditions used in this study. Therefore, the present findings do not establish that ATRA regulates VEGF through an HIF-1α-independent mechanism; rather, they indicate that lower intracellular VEGF measurements occurred without a parallel detectable change in intracellular HIF-1α under these experimental conditions.

ASA enhances the activation of JNK, ERK, and p38 MAPK in PC-9 lung cancer cells, suggesting that low-dose aspirin may exert context-dependent effects through the MAPK pathway [23]. Previous studies have demonstrated that ASA pretreatment can inactivate ERK1/2 and increase TRAIL-mediated caspase activation in HeLa human CC cell lines [16]. Additionally, ASA has been reported to inhibit AKT and ERK1/2 signaling in both endometrial stem cells [24] and CC cell lines [17], findings that are consistent with reduced survival-associated signaling and suppression of cellular proliferation. Consistent with these reports, our results indicate that ASA-containing treatments were associated with lower total ERK1/2 and phospho-AKT (Ser473) abundance in HeLa CC cells while favoring p38 MAPK/JNK-associated stress-marker increases. However, because apoptosis-specific endpoints such as caspase activation, PARP cleavage, Annexin V staining, or TUNEL analysis were not assessed in the present study, these protein-abundance changes should be interpreted as evidence of altered survival- and stress-associated measurements rather than direct proof of apoptosis induction. Notably, co-treatment with ASA and ATRA produced the lowest total ERK1/2 and phospho-AKT (Ser473) abundance values among the tested groups and was accompanied by the lowest measured cell viability. This combined-treatment profile suggests that ATRA and ASA may jointly alter selected survival-associated protein measurements in HeLa CC cells, although direct apoptosis assays would be required to determine whether apoptotic cell death occurred under these experimental conditions.

Consistent with previous research linking aspirin, COX-related biology, and angiogenesis-associated pathways, this study found lower intracellular VEGF concentrations in HeLa cell lysates after ASA-containing treatments. Aspirin has been reported to inhibit cancer metastasis and angiogenesis by targeting heparanase [25], and COX-2-related signaling has been linked to VEGF-dependent tumor-associated angiogenesis [13]. It has also been observed that a COX-2 selective inhibitor reduces HIF-1α expression through both COX-2-dependent and COX-2-independent mechanisms [21]. However, unlike those prior findings, ASA treatment did not significantly alter intracellular HIF-1α concentrations in the present study. Therefore, while ASA may influence VEGF-associated biology through COX-dependent or COX-independent pathways in other systems, the present data only support lower measured intracellular VEGF concentrations in HeLa cell lysates without detectable parallel HIF-1α modulation, and they do not establish reduced VEGF secretion or a specific anti-angiogenic mechanism.

The integrated computational findings complement the experimental observations at several levels. Cross-validated response modeling supports that viability patterns are structured across dose and exposure time, while bootstrap uncertainty demonstrates that the combination is not uniformly synergistic (Figure 7). STRING topology places AKT1 and MAPK proteins at the center of a dense hypothesis-driven network linking retinoid receptors, cyclooxygenases, TP53/JUN, and the HIF1A–VEGFA axis (Figure 8). This topology is compatible with lower total ERK1/2 and phospho-AKT (Ser473) abundance, higher p38 MAPK/JNK measurements, and lower intracellular VEGF concentrations, but it does not establish the direction or causal sequence of these changes. Target-prediction and docking results provide the clearest molecular support for established pharmacology: ATRA’s consensus target set contains multiple retinoid receptors (Figure 9), and its docking scores closely reproduce the native RARG ligand, supporting RARG as a plausible upstream anchor. ASA and salicylate predictions converge on PTGS1/PTGS2-related biology, while salicylate reproduces the noncovalent COX-2 reference score. However, ASA’s principal COX mechanism is covalent; therefore, its conventional docking poses should be described only as pre-reaction configurations. The markedly weaker VEGFR2 and AKT1 scores relative to native inhibitors do not support direct engagement of those proteins (Figure 10C,D). Patient-level TCGA-CESC results add disease relevance rather than treatment validation: VEGFA showed the most robust association because its adverse overall-survival relationship was observed using both median-split and continuous adjusted models, HIF1A was model-sensitive, and PTGS2 showed adverse overall-survival and progression-free-interval associations with proportional-hazards caution for progression-free interval (Figure 10B). Together, these data support VEGFA, HIF1A, and PTGS2 as clinically relevant components of the proposed network, but they do not demonstrate that ATRA or ASA modifies survival through these markers.

### Limitations

This study has several limitations. First, the experiments were conducted using a single established cervical cancer cell line, which may not fully reflect the biological heterogeneity of cervical cancer. Second, the findings are based on in vitro assays and therefore cannot directly predict in vivo pharmacokinetics, tumor microenvironment interactions, treatment tolerability, selectivity, or clinical efficacy. Third, although ERK1/2, phospho-AKT, p38 MAPK, JNK, HIF-1α, and VEGF were evaluated, additional upstream and downstream mediators of apoptosis, angiogenesis, and compensatory signaling were not comprehensively assessed. Importantly, apoptosis was not directly measured because caspase activation, PARP cleavage, Annexin V staining, TUNEL analysis, or comparable cell-death readouts were not performed. Therefore, apoptosis-related discussion should be interpreted only as inference from changes in survival- and stress-associated signaling measurements, not as direct evidence that ATRA, ASA, or their combination induced apoptotic cell death. Fourth, the Western blot analysis measured selected total or phosphorylated proteins but did not include paired phosphorylated and total measurements for all MAPK/AKT pathway components; therefore, these data cannot establish global pathway activation or inhibition. Fifth, VEGF and HIF-1α were measured in cell lysates under normoxic culture conditions. Secreted VEGF in conditioned medium, VEGF mRNA expression, independent normalization of VEGF to total protein or post-treatment cell number, and HIF-1α stabilization controls were not included. Accordingly, lower intracellular VEGF measurements should not be interpreted as direct evidence of reduced VEGF secretion, angiogenesis inhibition, or an HIF-1α-independent mechanism. Sixth, the computational component was intended as supporting mechanistic contextualization only. Standard docking does not model the covalent acetylation mechanism by which aspirin inhibits COX enzymes, and Vina scores should not be interpreted as experimental binding free energies. Seventh, the study did not include animal models, patient-derived cells, or normal cervical epithelial controls, which would be useful for evaluating selectivity and translational relevance.

## 4. Materials and Methods

### 4.1. Study Design

This in vitro experimental study evaluated the effects of ATRA, ASA, and their combination on HeLa cervical cancer cells. Cell viability was assessed by MTT assay after 24, 48, and 72 h of treatment, while pathway-related protein measurements and angiogenesis-associated markers were examined after 48 h using Western blotting and ELISA, respectively. All experiments were performed under standard normoxic culture conditions. Biological samples were considered the experimental units for statistical analysis; technical replicates were averaged before analysis and were not treated as independent biological observations.

### 4.2. Cell Culture

The HeLa cell line (human cervical adenocarcinoma, Homo sapiens) used in this study was obtained from the American Type Culture Collection (ATCC, Manassas, VA, USA; Cat. No. CCL-2™) in 2024. No additional cell-line authentication assay, such as STR profiling, was performed in our laboratory. HeLa cells between passages 1 and 10 were used for all experiments. Prior to experimentation, the cell line was tested for mycoplasma contamination using the LookOut^®^ One-Step Mycoplasma PCR Detection Kit (Merck KGaA, Darmstadt, Germany), according to the manufacturer’s instructions, and the results confirmed that the cells were mycoplasma-free. HeLa cells were cultured in Dulbecco’s modified Eagle’s medium (DMEM) supplemented with 10% FBS and 100 units/mL penicillin/streptomycin. Cells were maintained at 37 °C in a humidified atmosphere of 95% air and 5% CO_2_ under standard normoxic culture conditions. ATRA and ASA were initially dissolved in ethanol to prepare stock solutions, which were stored at −80 °C until use. Prior to treatment, stock solutions were diluted in culture medium to obtain final concentrations of 2.5–100 μM for ATRA and 0.25–10 mM for ASA. The control group received vehicle-matched culture medium containing the same final ethanol concentration as the treatment groups.

### 4.3. MTT Assay

The MTT assay was used to determine fitted half-maximal inhibitory concentration (IC_50_) estimates after HeLa cells were seeded at 1 × 10^4^ cells/well in 96-well plates and treated with increasing doses of ATRA (2.5–100 μM) and ASA (0.25–10 mM) for 24, 48, and 72 h. Each condition was analyzed using six technical replicate wells within each independent experiment. Measurements from technical replicates were averaged before statistical analysis and were not treated as independent biological observations. After treatment, the medium was removed, and 20 μL of MTT solution (5 mg/mL) and 100 μL of DMEM were added to each well. After incubation for 3 h, the medium was removed, and 100 μL of DMSO was added to dissolve the formazan crystals. After approximately 5 min, absorbance was measured at 570 nm using a microplate reader (Multiskan GO; Thermo Fisher Scientific, Waltham, MA, USA). IC_50_ values were calculated using GraphPad Prism software (version 9, La Jolla, CA, USA) and interpreted as fitted estimates within the tested concentration ranges and assay conditions. Treatment duration was initially evaluated at 24, 48, and 72 h; based on the fitted IC_50_ values, the 48 h time point was selected for subsequent Western blot and ELISA experiments because it provided a clear reduction in cell viability and a substantial treatment response while avoiding longer 72 h exposure. Results were expressed as the percentage of viable cells relative to vehicle-matched control cells.

### 4.4. Experimental Groups

Experimental groups were defined as control, ATRA, ASA, and ATRA + ASA. For experiments performed in 6-well plates, cells were seeded at 3 × 10^5^ cells/well. Twenty-four hours after seeding, the culture medium was replaced with fresh vehicle- or treatment-containing medium. The control group received vehicle-matched culture medium containing the same final ethanol concentration as that used in the treatment conditions, whereas the treatment groups received medium containing ATRA, ASA, or the ATRA + ASA combination. Cells were incubated for 48 h under standard normoxic conditions at 37 °C in a humidified atmosphere containing 5% CO_2_ and 95% air. No hypoxic exposure or HIF-1α-stabilizing agent, such as CoCl_2_, DMOG, or another hypoxia-mimetic treatment, was used. After 48 h of treatment, cell lysates for ELISA analysis were prepared using 1× RIPA lysis buffer and a protease inhibitor cocktail set (Merck KGaA, Darmstadt, Germany), as previously described [26]. Separate cell pellets for Western blot analysis were processed using ProtinEx Total Protein Extraction Solution, as described below. Cell lysates were stored at −80 °C until analysis.

### 4.5. Biochemical Analysis

#### 4.5.1. Assessment of HIF-1α and VEGF in Cell Lysates

Intracellular HIF-1α and VEGF concentrations in HeLa cell lysates were measured using commercially available sandwich ELISA kits (Elabscience Human HIF-1α ELISA Kit, Cat. No. E-EL-H6066; Human VEGF ELISA Kit, Cat. No. E-EL-H0111; Wuhan, China) according to the manufacturer’s protocols. All samples were analyzed in duplicate under identical laboratory conditions, and duplicate readings were averaged for each independently prepared sample. VEGF and HIF-1α measurements represent intracellular concentrations in cell lysates rather than secreted concentrations in conditioned medium. VEGF concentrations were reported as pg/mL of lysate and were not independently normalized to total protein, total cell number, or viable-cell number after treatment. VEGF mRNA expression and secreted VEGF concentrations in conditioned medium were not evaluated. For HIF-1α, the intra-assay and inter-assay coefficients of variation were 8.8% and 9.5%, respectively, and the analytical sensitivity was 37.5 pg/mL. For VEGF, the intra-assay coefficient of variation was less than 4.9%, the inter-assay coefficient of variation was less than 10%, and the analytical sensitivity was 18.75 pg/mL. A separate biological positive-control condition for HIF-1α induction was not included; therefore, the absence of significant HIF-1α differences should be interpreted only under the normoxic conditions examined.

#### 4.5.2. Western Blot Analysis

The abundance of total ERK1/2, phospho-AKT1/2/3 (Ser473), total p38 MAPK, and total JNK1/2/3 was determined by Western blot analysis using specific primary antibodies, with β-actin used as the loading control. Total AKT, phospho-ERK1/2, phospho-p38 MAPK, and phospho-JNK1/2/3 were not measured as part of the original experimental design; therefore, p-AKT/AKT, p-ERK/ERK, p-p38/p38, and p-JNK/JNK ratios could not be calculated. Consequently, the Western blot findings were interpreted as treatment-associated changes in selected measured protein abundance rather than definitive evidence of MAPK/AKT pathway activation or inhibition. Western blotting included protein extraction, protein quantification, sample preparation, SDS-PAGE, electrotransfer, membrane blocking, primary and secondary antibody incubation, chemiluminescent signal detection, and densitometric analysis.

Cells were washed with 1 mL of cold 1× PBS and centrifuged at 2500 rpm for 3 min. The pellet was resuspended in 1 mL of ProtinEx Total Protein Extraction Solution (GeneAll Biotechnology, Seoul, Republic of Korea; Cat. No. 701-001) supplemented with 10 µL protease inhibitor cocktail. After incubation on ice for 10 min, samples were centrifuged at 16,000 rpm for 15 min at 4 °C. The supernatant was collected for further analysis. Total protein concentration was determined using the Bradford Protein Assay Kit (ABP Biosciences, Beltsville, MD, USA; Cat. No. P010) according to the manufacturer’s instructions. Protein samples were mixed with 5× reducing SDS sample buffer (GeneAll Biotechnology, Seoul, Republic of Korea; Cat. No. 751-001), diluted with distilled water to a final volume of 20 µL, and denatured at 95 °C for 10 min.

Equal amounts of total protein were loaded onto 4–12% Bis-Tris gradient gels (Invitrogen, Thermo Fisher Scientific, Waltham, MA, USA; Cat. No. NP0321) and separated by SDS-PAGE using MOPS running buffer (1×; Invitrogen, Thermo Fisher Scientific; Cat. No. NP0005). Protein concentrations were determined using the Bradford assay, and the volume required for each sample was calculated individually to obtain an equal target protein load of 35 µg per lane. Gels were run initially at 80 V for 10 min, followed by 220 V for 50 min. Proteins were transferred onto nitrocellulose membranes using the iBlot 2 Dry Blotting System (Invitrogen, Thermo Fisher Scientific) with iBlot Transfer Stacks (NC). The transfer was carried out using program P3 for 7 min.

Membranes were blocked in 5% BSA in PBS-T containing 0.1% Tween-20 for 1 h at room temperature. Primary antibodies diluted in blocking buffer were incubated with membranes overnight at 4 °C with gentle agitation. The following rabbit primary antibodies from Affinity Biosciences (Cincinnati, OH, USA) were used: β-actin (Cat. No. AF7018; 1:3000), total JNK1/2/3 (Cat. No. AF6318; 1:500), total p38 MAPK (Cat. No. AF6456; 1:500), phospho-AKT1/2/3 (Ser473) (Cat. No. AF0016; 1:500), and total ERK1/2 (Cat. No. AF0155; 1:1000). Manufacturer-reported suitability for Western blotting in human samples was confirmed; no additional in-house antibody validation was performed. The next day, membranes were equilibrated to room temperature and incubated for an additional 2 h on a shaker. After primary incubation, membranes were washed three times with PBS-T for 5 min each. Subsequently, HRP-conjugated goat anti-rabbit IgG (H+L) secondary antibody (Affinity Biosciences; Cat. No. S0001; 1:3000) diluted in 5% BSA-PBS-T was applied for 1 h at room temperature. Membranes were then washed three times with PBS-T. Protein bands were visualized using WesternBright ECL substrate (Advansta, San Jose, CA, USA; Cat. No. K-12045-D50) and detected with the G:BOX Chemi XRQ imaging system (Syngene, Cambridge, UK).

Densitometric analysis was performed using ImageJ (v1.54p, U.S. National Institutes of Health, Bethesda, MD, USA) after local background correction. Background-corrected target-band intensities were normalized to the corresponding β-actin band intensity and expressed relative to the mean value of the vehicle-matched control group, which was assigned a value of 1.00. Further data processing was performed in Microsoft Excel. Figure 4 presents quantitative densitometric analyses, while Supplementary Figure S2 provides the available archived images for all six samples per treatment group. Original uncropped, full-length blots containing molecular-weight markers were not retained, and exposure settings and formal linear-range testing were not available; these limitations are explicitly acknowledged in the manuscript.

### 4.6. Statistical Analysis

Statistical analyses were performed using Jamovi software (version 2.3.21), and IC_50_ values for the MTT assay were calculated using GraphPad Prism software (version 9, La Jolla, CA, USA). Data are presented as the mean ± standard deviation (SD). MTT experiments were independently repeated three times unless otherwise specified, representing independent biological replicates. For MTT assays, six technical replicate wells were used within each independent experiment, and technical replicate measurements were averaged before statistical testing. For the concentration × time MTT analysis, a two-factor ANOVA framework was used to evaluate the main effects of concentration and treatment duration and the concentration × time interaction separately for ATRA and ASA; no repeated-measures structure was applied because measurements at each concentration–time condition were obtained from separate wells. For Western blot and ELISA experiments, *n* = 6 refers to six independently prepared cell-culture samples per experimental group; technical replicate measurements, including duplicate ELISA readings, were averaged for each sample and were not treated as independent observations. The biological sample was considered the unit of analysis. Comparisons among the control, ATRA, ASA, and ATRA + ASA groups were then performed using one-way analysis of variance (ANOVA), followed by Holm-corrected post hoc multiple-comparison testing when appropriate. For pathway-related protein measurements and ELISA-based measurements, a two-factor ANOVA model was used to assess the main effects of ATRA and ASA and the ATRA × ASA interaction for each marker. Post hoc pairwise comparisons were adjusted using the Holm correction to control for multiple testing. To evaluate whether the combined-treatment response reflected independent single-agent effects, observed ATRA + ASA values were compared with expected independent-effect responses calculated from the corresponding ATRA-only and ASA-only effects after normalization to the control group. Bliss expected inhibition was calculated as E_A_ + E_B_ − E_A_E_B_, where E_A_ and E_B_ represent the fractional inhibitions produced by ATRA and ASA alone at the corresponding concentrations. Bliss excess inhibition was defined as observed combined inhibition minus Bliss expected inhibition. For fixed-ratio interaction interpretation, Bliss excess values >+5 percentage points were considered synergistic, values from −5 to +5 percentage points additive, and values <−5 percentage points antagonistic. Chou–Talalay combination index and Loewe additivity values <0.90 were classified as synergy, 0.90–1.10 as additivity, and >1.10 as antagonism. Western blot protein-abundance values were normalized to β-actin, which served as the loading control. All tests were two-sided, and statistical significance was accepted at *p* < 0.05.

### 4.7. Computational Target Prediction, Survival Analysis, and Molecular Docking

Computational analyses were performed as supporting mechanistic context and were not interpreted as evidence of treatment efficacy. Nonzero-dose MTT measurements were normalized to matched time-specific controls, and candidate dose–time response surfaces were fitted on the logit-viability scale and compared using leave-one-condition-out cross-validation. Uncertainty in Bliss excess inhibition was estimated using 20,000 nonparametric bootstrap resamples of the six combination replicates, matched single-agent replicates, and pooled 48 h control observations, with prespecified thresholds of >+5 percentage points for synergy, −5 to +5 points for additivity, and <−5 points for antagonism [27,28]. A hypothesis-driven 14-protein STRING v11.5 network was constructed using retinoid receptors, cyclooxygenases, MAPK/AKT signaling proteins, TP53, HIF1A, and VEGFA; associations with combined score ≥0.400 were retained, centrality metrics were calculated, and enrichment was evaluated with STRING false-discovery-rate correction [29]. VEGFA was retained in the topology analysis but excluded from enrichment because it resolved incorrectly to COL18A1. Consensus target prediction was conducted by intersecting SwissTargetPrediction hits meeting probability ≥0.10 with significant human SEA targets meeting *p* ≤ 0.05 for ATRA, ASA, and salicylate [30,31]. TCGA-CESC overall survival and progression-free interval associations were evaluated using median-split Kaplan–Meier analysis and adjusted Cox proportional-hazards models with expression modeled per standard-deviation increase and adjustment for age, FIGO stage III/IV, and histology, using publicly available TCGA/GDC cervical cancer data [32,33]. Molecular docking was performed for RARG, COX-2, VEGFR2, and AKT1 using structure-specific search boxes, exhaustiveness 32, and relevant ligand protonation states. Each protocol was validated by native-ligand redocking using a prespecified heavy-atom RMSD threshold of ≤2.0 Å, and Vina scores were interpreted only relative to native ligands within the same protein system rather than as experimental binding free energies [34]. Standard docking was treated as a noncovalent pre-reaction model and was not considered capable of reproducing aspirin-mediated acetyl transfer to COX-2 Ser530. PharmMapper results were unavailable, and the planned 100 ns MD/MM-PBSA workflow was not executed; these analyses are therefore reported as unavailable or planned rather than confirmatory evidence.

## 5. Conclusions

Taken together, these in vitro findings indicate that ATRA and ASA reduce HeLa cell viability and are associated with lower total ERK1/2 and phospho-AKT (Ser473) abundance, together with lower intracellular VEGF concentrations without detectable changes in intracellular HIF-1α under normoxic culture conditions. The combined treatment produced the lowest total ERK1/2, phospho-AKT (Ser473), and intracellular VEGF measurements among the tested groups, and VEGF was the only measured marker showing a significant ATRA × ASA interaction. Because the interaction analyses were exploratory and were not based on a full pharmacological dose–response matrix with matched combination-control conditions, these findings should be interpreted as stronger measured responses rather than definitive evidence of drug synergy. They also do not establish global MAPK/AKT pathway inhibition, reduced VEGF secretion, angiogenesis inhibition, or an HIF-1α-independent regulatory mechanism. The computational analyses support pathway-level coherence across retinoid receptor, COX/prostaglandin, MAPK/AKT, hypoxia, and angiogenic modules, reinforce the clinical relevance of VEGFA, HIF1A, and PTGS2 in TCGA-CESC, and provide docking support for RARG–ATRA and COX-2–salicylate pose plausibility. Conversely, they do not establish direct VEGFR2 or AKT1 engagement by ASA or salicylate and should not be considered proof of therapeutic efficacy. Because this study was limited to an in vitro HeLa model with supporting computational analyses, validation in additional experimental systems, preclinical models, external cohorts, and clinical studies is required before translational conclusions can be drawn. These findings identify total ERK1/2, phospho-AKT (Ser473), intracellular VEGF, and computationally prioritized retinoid/inflammatory signaling targets as candidate downstream markers or associated pathways for future studies of combined ATRA and ASA exposure in cervical cancer cell-line models.

## Figures and Tables

**Figure 1:**
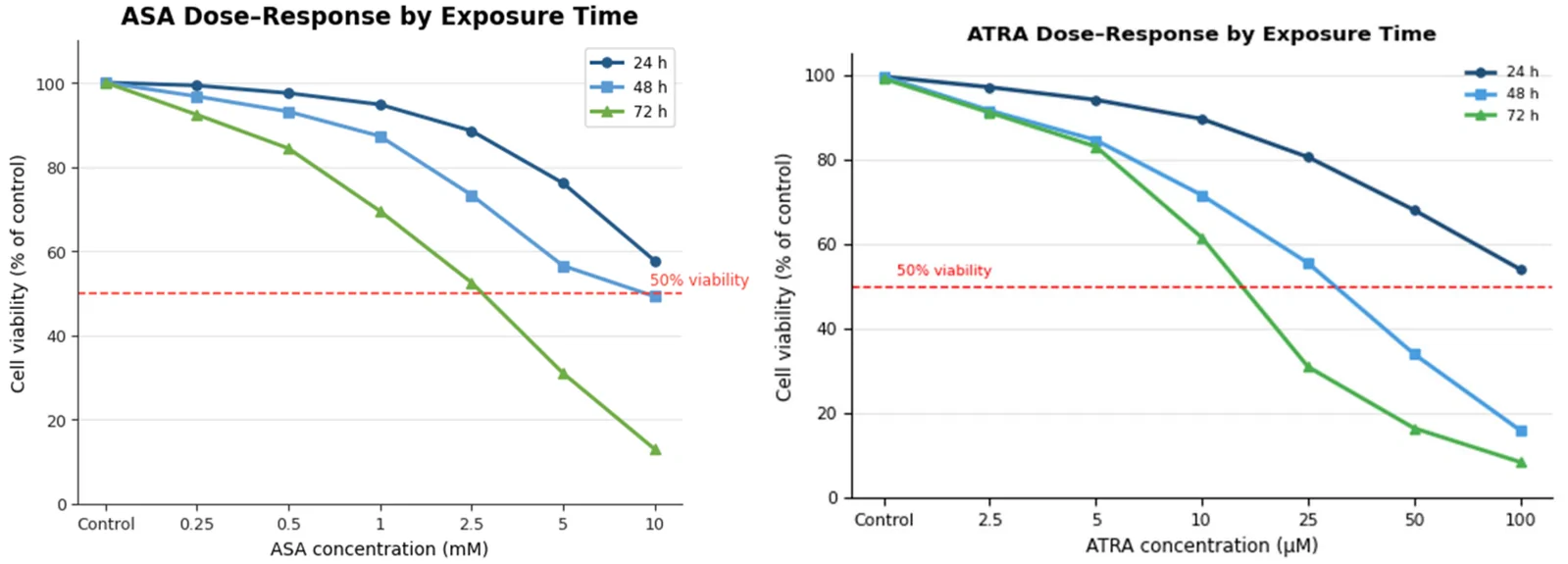
Dose- and time-dependent effects of ATRA and ASA on HeLa cell viability as measured by MTT assay. HeLa cells were treated with increasing concentrations of ATRA or ASA for 24, 48, and 72 h. The left panel shows ATRA-treated cells, and the right panel shows ASA-treated cells. The *x*-axis represents drug concentration, and the *y*-axis represents cell viability relative to vehicle-matched control cells based on absorbance at 570 nm. Data are presented as the mean ± SD; replicate structure is described in Section 4.6. ATRA: all-trans retinoic acid; ASA: acetylsalicylic acid.

**Figure 2:**
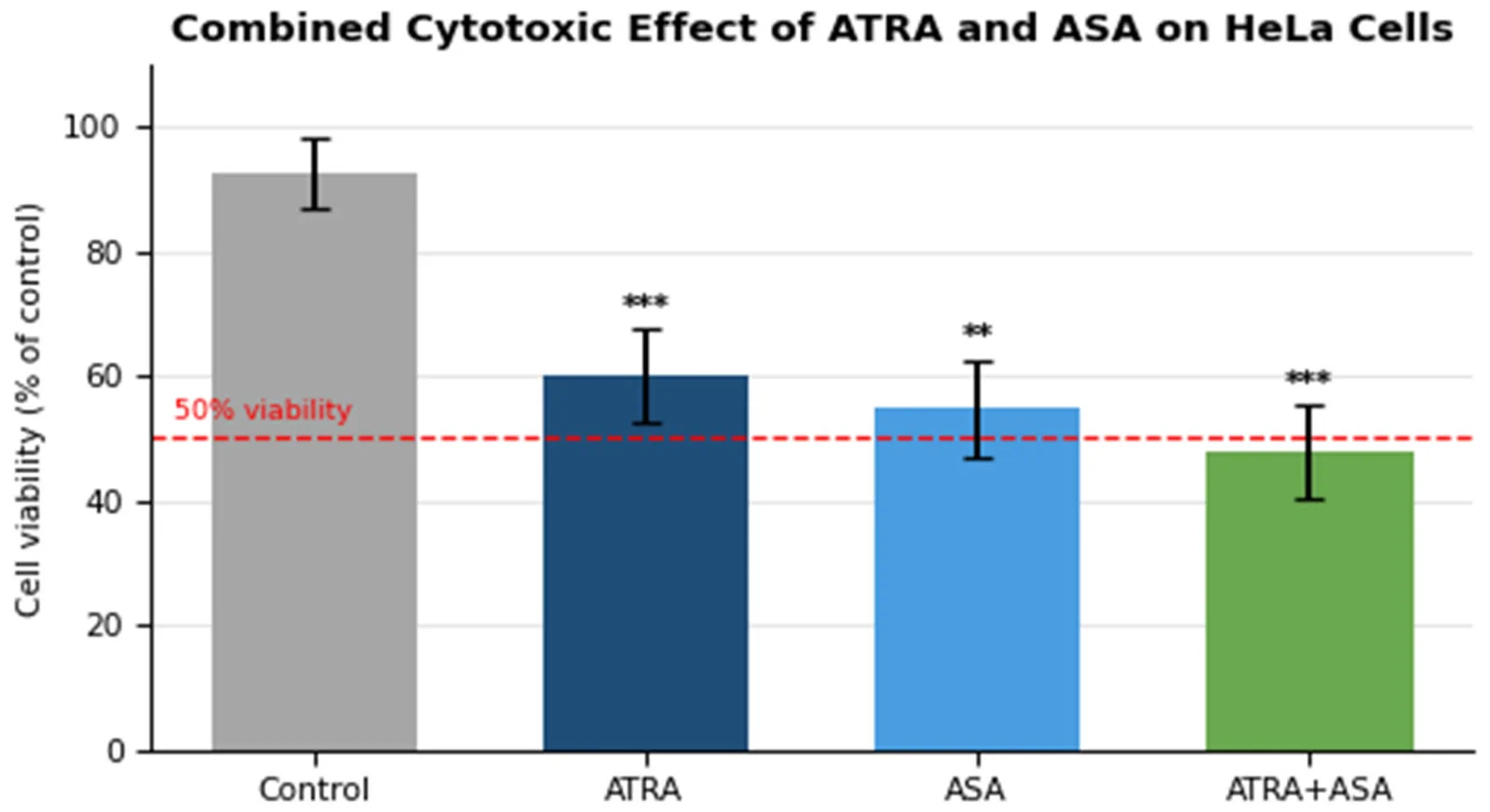
Effects of ATRA, ASA, and combined ATRA + ASA treatment on HeLa cell viability at 48 h, expressed as a percentage of vehicle-matched control cells. The *x*-axis shows the treatment groups, including control, ATRA, ASA, and ATRA + ASA, and the *y*-axis shows cell viability relative to vehicle-matched control cells. Data are presented as the mean ± SD; replicate structure is described in Section 4.6. ATRA: all-trans retinoic acid; ASA: acetylsalicylic acid. Significance is shown versus Control: ** *p* < 0.01; *** *p* < 0.001.

**Figure 3:**
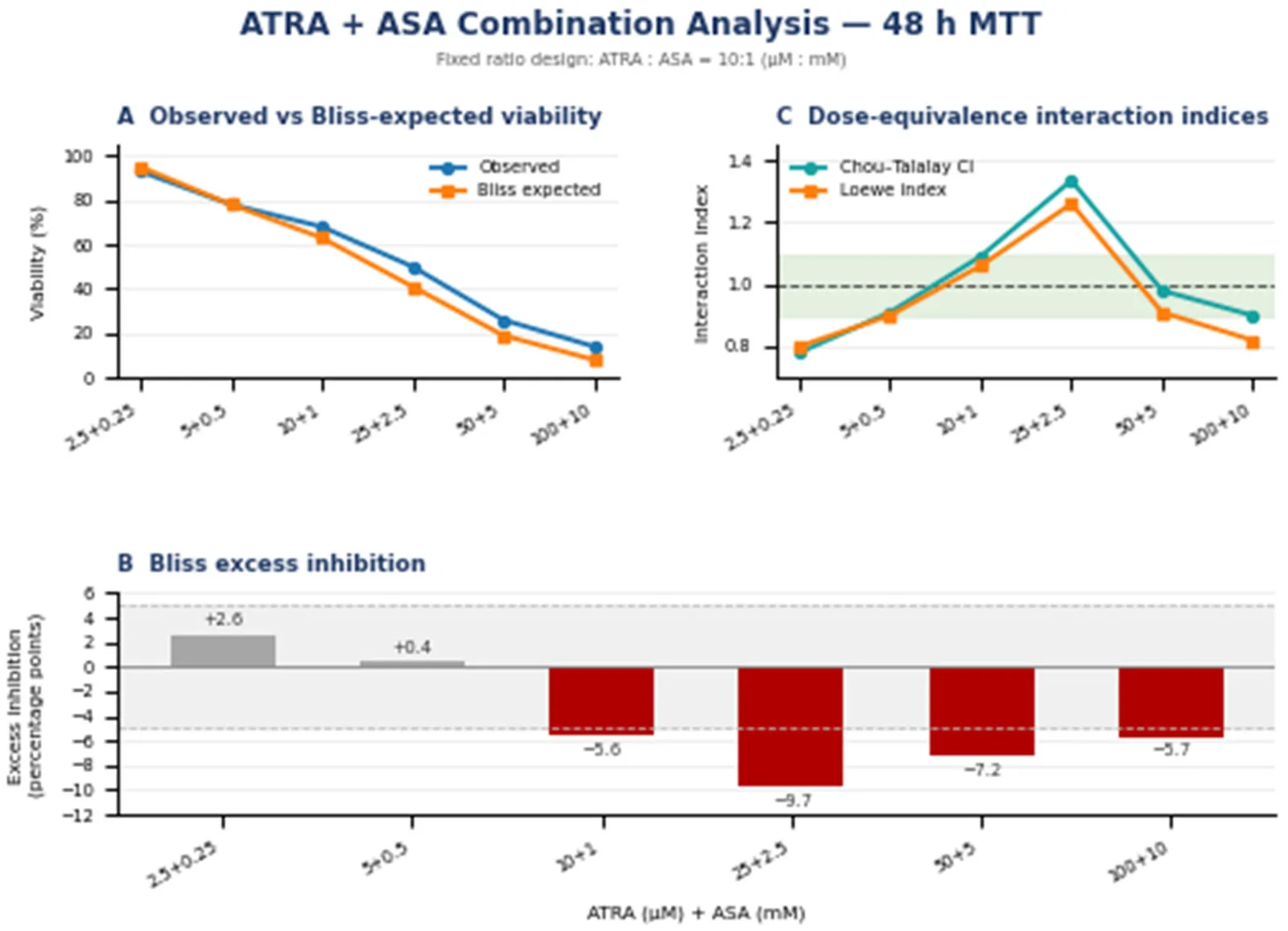
Fixed-ratio interaction analysis between ATRA and ASA in HeLa cells at 48 h. (**A**) Observed cell viability and the Bliss-independent expectation. (**B**) Bliss excess inhibition, where values greater than +5 percentage points indicate synergy, values from −5 to +5 indicate additivity, and values less than −5 indicate antagonism. (**C**) Chou–Talalay combination index (CI) and Loewe additivity index, where values less than 0.90 indicate synergy, 0.90–1.10 indicate additivity, and greater than 1.10 indicate antagonism. Treatments followed a fixed 10:1 ATRA (µM):ASA (mM) ratio. Because matched combination-control conditions and a full pharmacological dose–response matrix were not available, this analysis should be interpreted as exploratory interaction assessment rather than definitive pharmacological synergy modeling.

**Figure 4:**
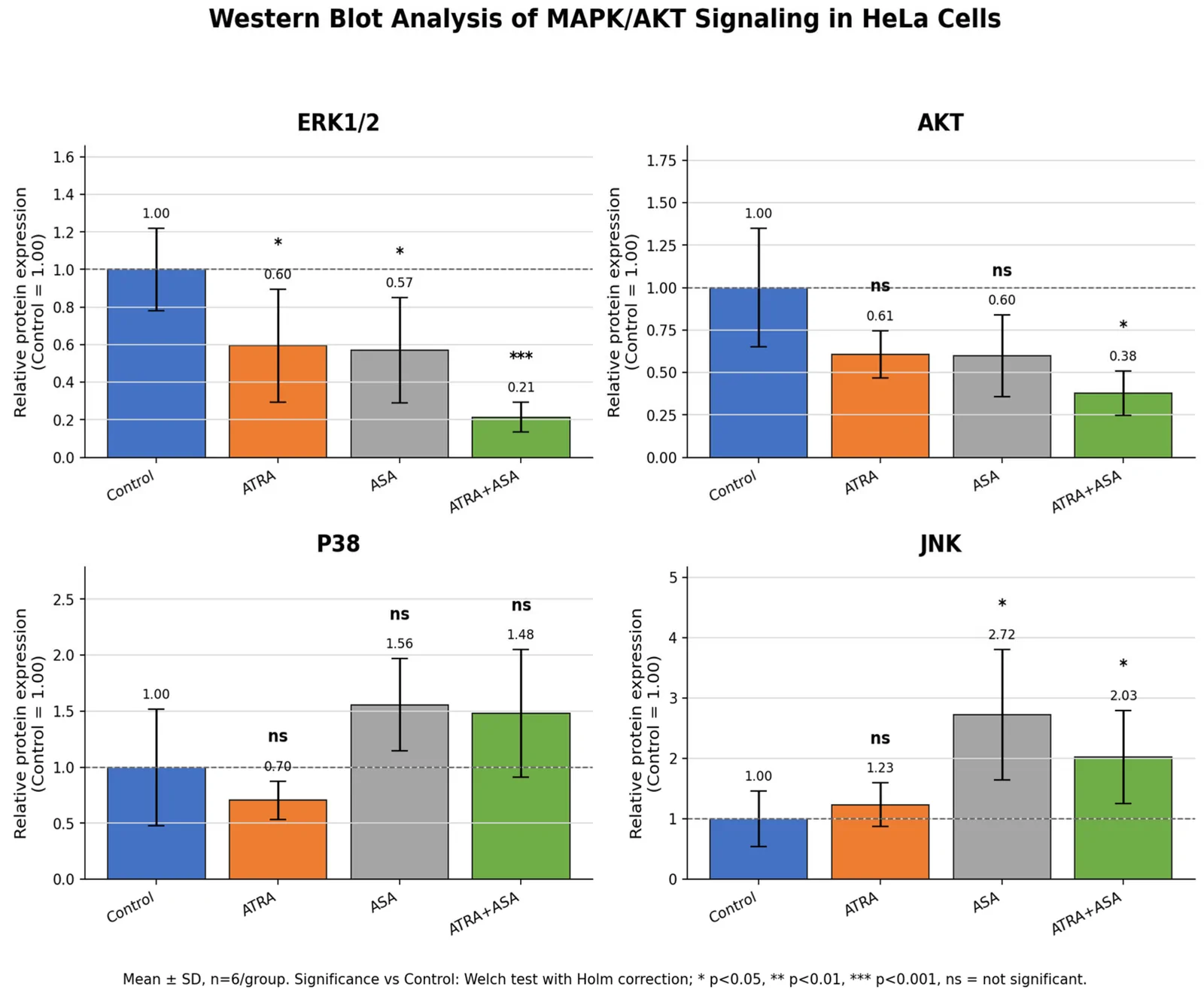
Effects of ATRA, ASA, and combined ATRA + ASA treatment on selected MAPK/AKT-related protein abundance in HeLa cells. The upper panels show total ERK1/2 and phospho-AKT (Ser473), and the lower panels show total p38 MAPK and total JNK. The x-axis shows the treatment groups, and the y-axis shows relative protein abundance presented as arbitrary units after normalization to β-actin. Data are presented as the mean ± SD (*n* = 6). ATRA: all-trans retinoic acid; ASA: acetylsalicylic acid; JNK: c-Jun N-terminal kinase; MAPK: mitogen-activated protein kinase. * *p* < 0.05; *** *p* < 0.001. Because paired phosphorylated/total measurements were not available for all proteins, the results should not be interpreted as comprehensive pathway activation or inhibition.

**Figure 5:**
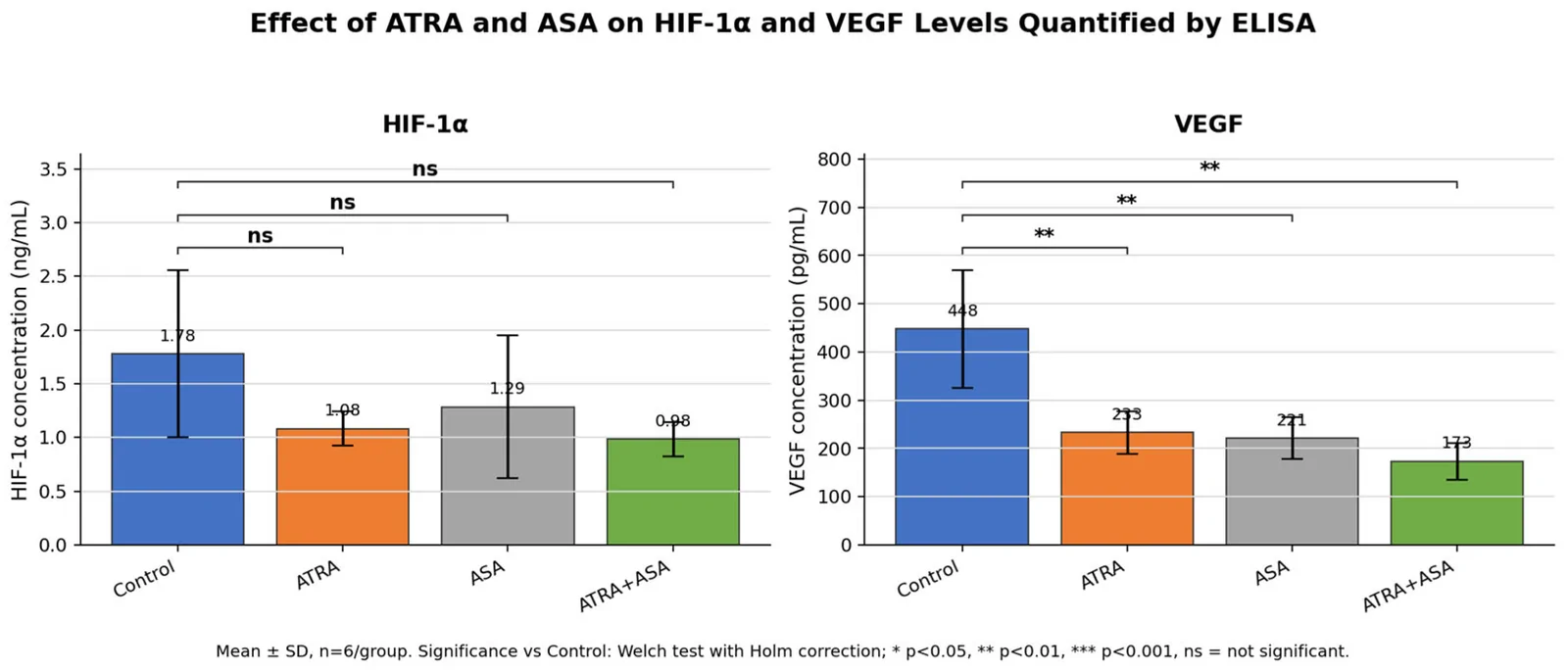
Effects of ATRA, ASA, and their combination on intracellular HIF-1α and VEGF concentrations in HeLa cell lysates as determined by ELISA. The figure shows HIF-1α and VEGF measurements across treatment groups. The x-axis shows the treatment groups, and the y-axis shows protein concentration according to the ELISA assay output, reported as ng/mL for HIF-1α and pg/mL for VEGF. Data are presented as mean ± SD (*n* = 6). ATRA: all-trans retinoic acid; ASA: acetylsalicylic acid; HIF-1α: hypoxia-inducible factor 1α; VEGF: vascular endothelial growth factor. * p < 0.05; ** *p* < 0.01. VEGF measurements represent cell-lysate concentrations and should not be interpreted as secreted VEGF measurements.

**Figure 6:**
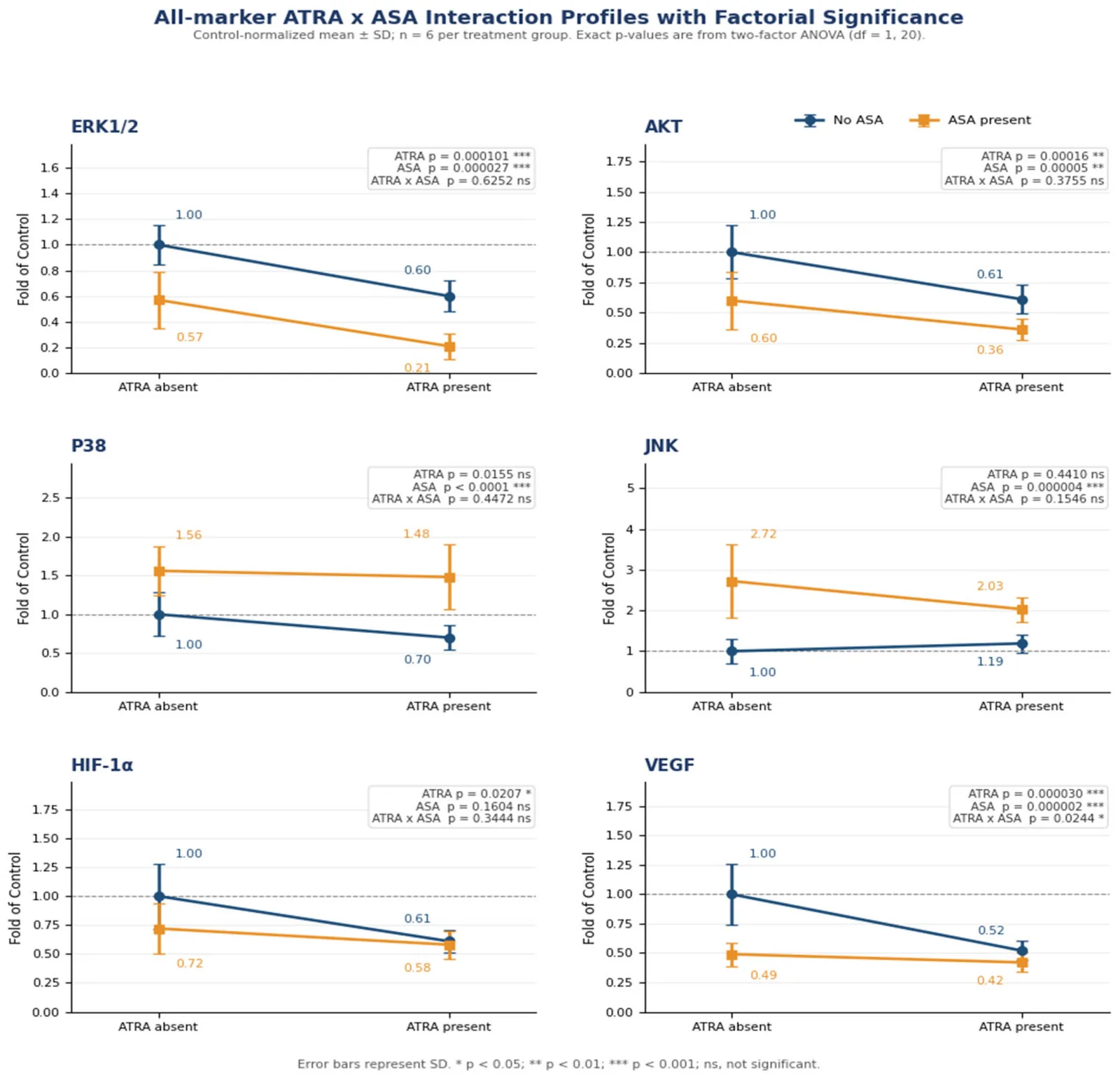
ATRA × ASA interaction profiles across total ERK1/2, phospho-AKT (Ser473), total p38 MAPK, total JNK, HIF-1α, and VEGF. Values are normalized to each marker’s control mean and shown as mean ± SD for the 2 × 2 factorial design. Two-factor ANOVA tested ATRA main effects, ASA main effects, and ATRA × ASA interactions. Total ERK1/2 and phospho-AKT (Ser473) were influenced by both main effects, p38 MAPK and JNK were predominantly ASA-driven, HIF-1α showed no significant treatment-dependent reduction, and VEGF was the only marker with a significant ATRA × ASA interaction.

**Figure 7:**
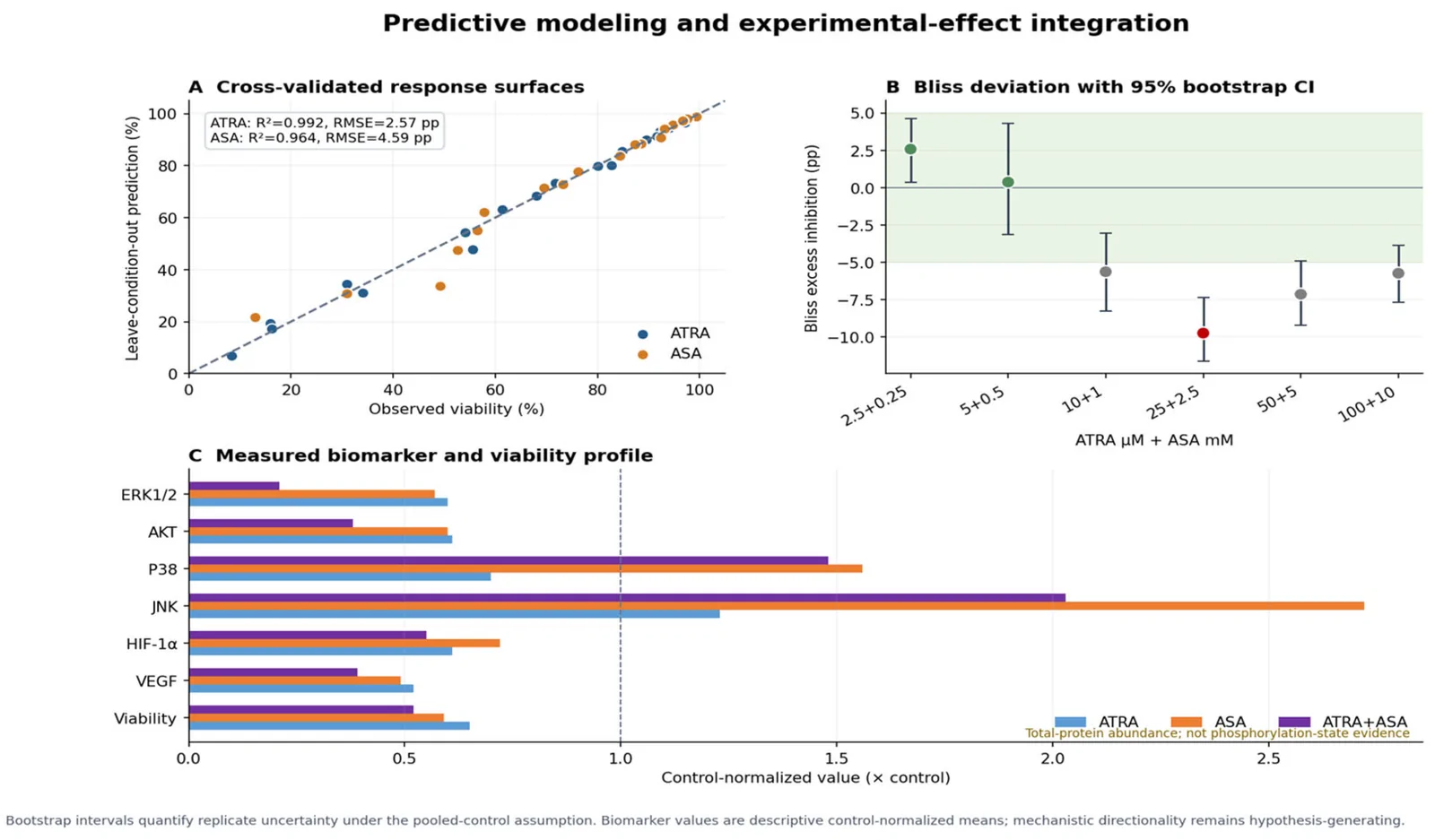
Predictive modeling and experimental-effect integration. (**A**) Cross-validated response-surface performance for ATRA and ASA, showing agreement between observed viability and leave-condition-out predictions. (**B**) Bliss excess inhibition with 95% bootstrap confidence intervals across fixed-ratio ATRA + ASA combinations. The shaded region indicates the prespecified additive interval from −5 to +5 percentage points. (**C**) Descriptive control-normalized biomarker and viability profile across ATRA, ASA, and ATRA + ASA treatment groups. Bootstrap intervals quantify replicate uncertainty under the pooled-control assumption, and biomarker profiles should be interpreted as descriptive and hypothesis-generating.

**Figure 8:**
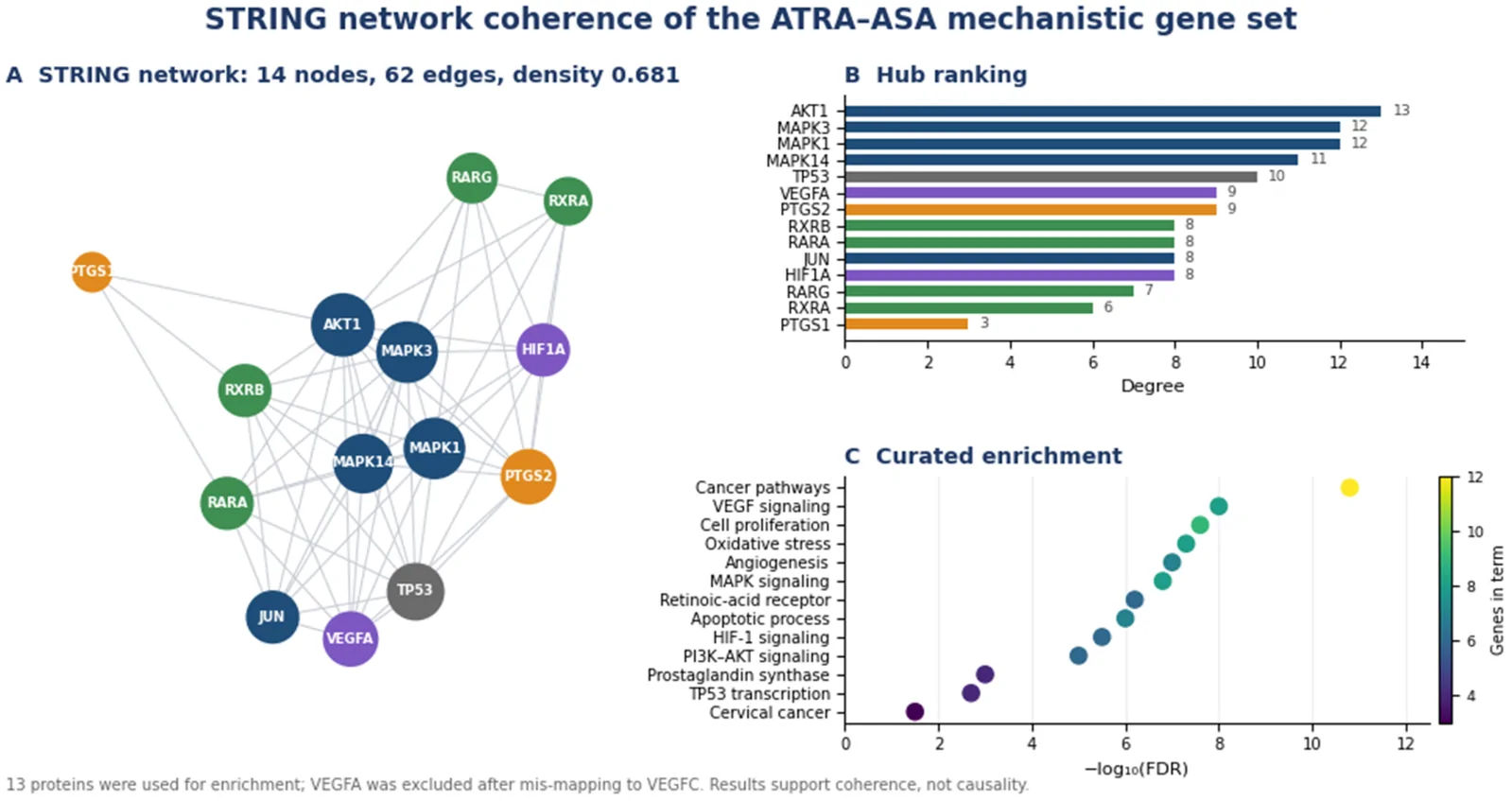
STRING network topology and functional enrichment of the proposed ATRA–ASA mechanism. (**A**) Hypothesis-driven STRING network comprising 14 nodes and 62 associations, with density = 0.681. (**B**) Hub ranking by degree centrality, showing AKT1, MAPK1, MAPK3, and MAPK14 among the highest-ranking nodes. (**C**) Curated functional enrichment terms linking retinoic-acid receptor, COX/prostaglandin, MAPK/AKT, oxidative-stress, HIF-1, VEGF, angiogenesis, and cervical-cancer-associated pathways. Thirteen proteins were used for enrichment; VEGFA was excluded after mis-mapping to COL18A1. STRING edges represent functional associations rather than experimentally confirmed physical interactions in HeLa cells.

**Figure 9:**
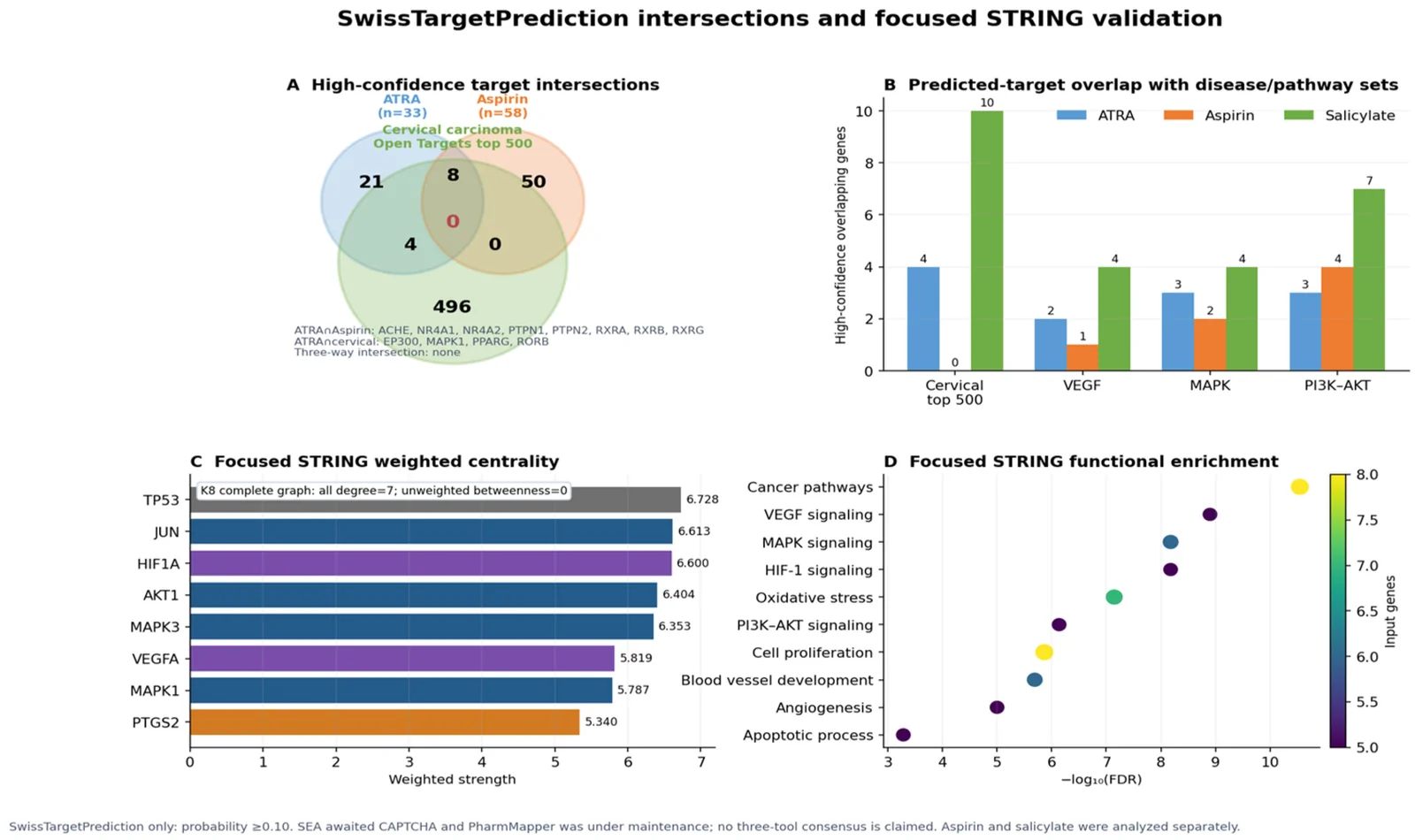
Compound-specific target prediction and pathway convergence. SwissTargetPrediction results and reference-set intersections are shown for ATRA, ASA, and salicylate. The updated primary analysis uses SwissTargetPrediction plus SEA consensus sets of 15, 31, and 38 predicted targets for ATRA, ASA, and salicylate, respectively. The figure summarizes overlap between compound-specific predicted targets and disease- or pathway-relevant target sets, including VEGF-, MAPK-, and PI3K–AKT-related categories. These predictions are hypothesis-generating and should not be interpreted as evidence of binding, potency, or treatment efficacy.

**Figure 10:**
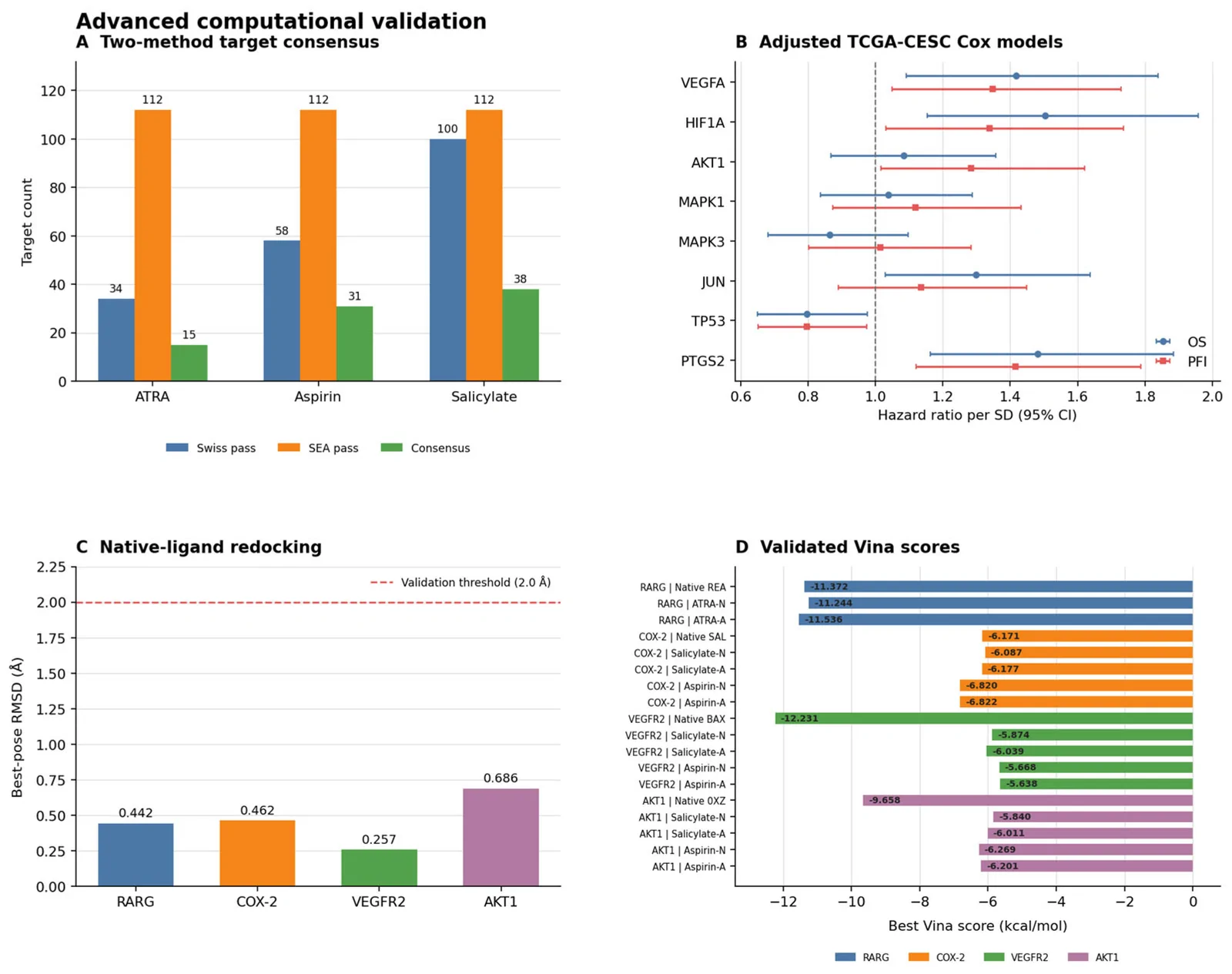
Four-panel computational summary of the proposed ATRA–aspirin mechanism. (**A**) SwissTargetPrediction and SEA consensus target counts/intersections for ATRA, ASA, and salicylate. (**B**) Adjusted Cox models showing overall survival (blue) and progression-free interval (red) associations for selected cervical-cancer-relevant targets in TCGA-CESC. (**C**) Native-ligand redocking validation, including RMSD values used to confirm docking-protocol reliability. (**D**) Comparative Vina docking-score summary for ATRA, ASA, salicylate, and corresponding native/reference ligands. Docking scores should be interpreted as docking-function outputs rather than experimental binding free energies, and the computational analyses should be regarded as supportive and hypothesis-generating rather than confirmatory evidence of treatment efficacy.

## Data Availability

Data is contained within the article and the Supplementary Materials.

## Supplementary Materials

The following supporting information can be downloaded at: https://www.mdpi.com/article/10.3390/ph19101556/s1: Supplementary Figure S1, GO and KEGG functional enrichment of the hypothesis-driven STRING protein set; Supplementary Figure S2, archived Western blot images for all six samples per treatment group; and Supplementary Table S1, numerical fixed-ratio ATRA + ASA interaction metrics, including Bliss excess inhibition, bootstrap confidence intervals, Chou–Talalay combination index, and Loewe additivity values.

## Abbreviations

The following abbreviations are used in this manuscript:

AKT	Protein kinase B
AKT1	AKT serine/threonine kinase 1
ANOVA	Analysis of variance
BSA	Bovine serum albumin
BH	Benjamini–Hochberg
ASA	Acetylsalicylic acid
ATRA	All-trans retinoic acid
COX	Cyclooxygenase
CI	Confidence interval or combination index, according to context
DMEM	Dulbecco’s modified Eagle’s medium
DMSO	Dimethyl sulfoxide
ELISA	Enzyme-linked immunosorbent assay
FDR	False discovery rate
FBS	Fetal bovine serum
ERK	Extracellular signal-regulated kinase
HIF-1α	Hypoxia-inducible factor 1 alpha
HR	Hazard ratio
JNK	c-Jun N-terminal kinase
MAPK	Mitogen-activated protein kinase
MM-PBSA	Molecular mechanics Poisson–Boltzmann surface area
PBS-T	Phosphate-buffered saline containing Tween-20
MTT	3-(4,5-dimethylthiazol-2-yl)-2,5-diphenyltetrazolium bromide
PTGS2	Prostaglandin-endoperoxide synthase 2
RAR	Retinoic acid receptor
RARG	Retinoic acid receptor gamma
RMSD	Root-mean-square deviation
RXR	Retinoid X receptor
SDS-PAGE	Sodium dodecyl sulfate–polyacrylamide gel electrophoresis
SEA	Similarity Ensemble Approach
STR	Short tandem repeat
TCGA-CESC	The Cancer Genome Atlas cervical squamous cell carcinoma and endocervical adenocarcinoma cohort
VEGFR2	Vascular endothelial growth factor receptor 2
Vina	AutoDock Vina docking score
VEGF	Vascular endothelial growth factor

## References

[1] Singh D., Vignat J., Lorenzoni V., Eslahi M., Ginsburg O., Lauby-Secretan B., Arbyn M., Basu P., Bray F., Vaccarella S. (2023). Global estimates of incidence and mortality of cervical cancer in 2020: A baseline analysis of the WHO Global Cervical Cancer Elimination Initiative. Lancet Glob. Health.

[2] Karnik M., Tulimilli S.V., Anantharaju P.G., Bettadapura A.D.S., Natraj S.M., Mohideen H.S., Dovat S., Sharma A., Madhunapantula S.V. (2026). An overview of the mechanisms of HPV-induced cervical cancer: The role of kinase targets in pathogenesis and drug resistance. Cancers.

[3] Bossler F., Hoppe-Seyler K., Hoppe-Seyler F. (2019). PI3K/AKT/mTOR signaling regulates the virus/host cell crosstalk in HPV-positive cervical cancer cells. Int. J. Mol. Sci..

[4] Nagai Y., Ambinder A.J. (2023). The promise of retinoids in the treatment of cancer: Neither burnt out nor fading away. Cancers.

[5] Hunsu V.O., Facey C.O.B., Fields J.Z., Boman B.M. (2021). Retinoids as chemo-preventive and molecular-targeted anti-cancer therapies. Int. J. Mol. Sci..

[6] Ni X., Hu G., Cai X. (2019). The success and the challenge of all-trans retinoic acid in the treatment of cancer. Crit. Rev. Food Sci. Nutr..

[7] Zhang X., Dai B., Zhang B., Wang Z. (2012). Vitamin A and risk of cervical cancer: A meta-analysis. Gynecol. Oncol..

[8] Helm C.W., Lorenz D.J., Meyer N.J., Rising W.W., Wulff J.L. (2013). Retinoids for preventing the progression of cervical intra-epithelial neoplasia. Cochrane Database Syst. Rev..

[9] Castro-Guijarro A.C., Vanderhoeven F., Mondaca J.M., Redondo A.L., Zoppino F.C.M., Fernandez-Muñoz J.M., Sanchez A.M., Flamini M.I. (2022). Combination treatment of retinoic acid plus focal adhesion kinase inhibitor prevents tumor growth and breast cancer cell metastasis. Cells.

[10] Ma Y.T., Li C., Shen Y., You W.H., Han M.X., Mu Y.F., Han F.-J. (2025). Mechanisms of the JNK/p38 MAPK signaling pathway in drug resistance in ovarian cancer. Front. Oncol..

[11] Cao Z., Liao Q., Su M., Huang K., Jin J., Cao D. (2019). AKT and ERK dual inhibitors: The way forward?. Cancer Lett..

[12] Finetti F., Paradisi L., Bernardi C., Pannini M., Trabalzini L. (2023). Cooperation between prostaglandin E2 and epidermal growth factor receptor in cancer progression: A dual target for cancer therapy. Cancers.

[13] Yoshida S., Amano H., Hayashi I., Kitasato H., Kamata M., Inukai M., Yoshimura H., Majima M. (2003). COX-2/VEGF-dependent facilitation of tumor-associated angiogenesis and tumor growth in vivo. Lab. Investig..

[14] Dovizio M., Tacconelli S., Sostres C., Ricciotti E., Patrignani P. (2012). Mechanistic and pharmacological issues of aspirin as an anticancer agent. Pharmaceuticals.

[15] Sankaranarayanan R., Kumar D.R., Altinoz M.A., Bhat G.J. (2020). Mechanisms of colorectal cancer prevention by aspirin—A literature review and perspective on the role of COX-dependent and -independent pathways. Int. J. Mol. Sci..

[16] Im S.R., Jang Y.J. (2012). Aspirin enhances TRAIL-induced apoptosis via regulation of ERK1/2 activation in human cervical cancer cells. Biochem. Biophys. Res. Commun..

[17] Xiang S., Sun Z., He Q., Yan F., Wang Y., Zhang J. (2010). Aspirin inhibits ErbB2 to induce apoptosis in cervical cancer cells. Med. Oncol..

[18] Dong D., Fu Y., Chen F., Zhang J., Jia H., Li J., Wang H., Wen J. (2021). Hyperoxia sensitizes hypoxic HeLa cells to ionizing radiation by downregulating HIF-1α and VEGF expression. Mol. Med. Rep..

[19] Kim M.S., Kim Y.K., Eun H.C., Cho K.H., Chung J.H. (2006). All-trans retinoic acid antagonizes UV-induced VEGF production and angiogenesis via the inhibition of ERK activation in human skin keratinocytes. J. Investig. Dermatol..

[20] Song G., Li Y., Zhang Z., Ren X., Li H., Zhang W., Wei R., Pan S., Shi L., Bi K. (2013). c-myc but not HIF-1α-dependent downregulation of VEGF influences the proliferation and differentiation of HL-60 cells induced by ATRA. Oncol. Rep..

[21] Zhong H., Willard M., Simons J. (2004). NS398 reduces hypoxia-inducible factor (HIF)-1α and HIF-1 activity: Multiple-level effects involving cyclooxygenase-2-dependent and -independent mechanisms. Int. J. Cancer.

[22] Lee J.W., Cho J.Y., Thuy P.X., Moon E.Y. (2022). HeLa cervical cancer cells are maintained by nephronophthisis 3-associated primary cilium formation via ROS-induced ERK and HIF-1α activation under serum-deprived normoxic condition. Int. J. Mol. Sci..

[23] Qian Y., Dai H., Li H. (2021). Low-doses of aspirin promote the growth of human PC-9 lung cancer cells through activation of the MAPK family. Exp. Ther. Med..

[24] Park S.R., Kim S.R., Min E.K., Oh B.C., Jung Y., Kim Y.H., Lee H.-Y. (2023). Unveiling the potential effects of acetylsalicylic acid: Insights into regeneration in endometrial stem cells. Cell Commun. Signal..

[25] Dai X., Yan J., Fu X., Pan Q., Sun D., Xu Y., Wang J., Nie L., Tong L., Shen A. (2017). Aspirin inhibits cancer metastasis and angiogenesis via targeting heparanase. Clin. Cancer Res..

[26] Dogan Z., Ergun D.D., Durmus S., Sahin H., Senturk G.E., Gelisgen R., Senyigit A., Uzun H. (2023). Empagliflozin and sacubitril/valsartan reverse methotrexate cardiotoxicity by repressing oxidative stress and hypoxia in heart embryonic H9c2 cardiomyocytes: The role of morphology of mitochondria observed on electron microscopy. Eur. Rev. Med. Pharmacol. Sci..

[27] Bliss C.I. (1939). The toxicity of poisons applied jointly. Ann. Appl. Biol..

[28] Demidenko E., Miller T.W. (2019). Statistical determination of synergy based on Bliss definition of drugs independence. PLoS ONE.

[29] Szklarczyk D., Gable A.L., Nastou K.C., Lyon D., Kirsch R., Pyysalo S., Doncheva N.T., Legeay M., Fang T., Bork P. (2021). The STRING database in 2021: Customizable protein-protein networks, and functional characterization of user-uploaded gene/measurement sets. Nucleic Acids Res..

[30] Daina A., Michielin O., Zoete V. (2019). SwissTargetPrediction: Updated data and new features for efficient prediction of protein targets of small molecules. Nucleic Acids Res..

[31] Wang Z., Liang L., Yin Z., Lin J. (2016). Improving chemical similarity ensemble approach in target prediction. J. Cheminform..

[32] Cancer Genome Atlas Research Network (2017). Integrated genomic and molecular characterization of cervical cancer. Nature.

[33] National Cancer Institute Genomic Data Commons GDC Data Portal: TCGA-CESC Project. https://portal.gdc.cancer.gov/.

[34] Trott O., Olson A.J. (2010). AutoDock Vina: Improving the speed and accuracy of docking with a new scoring function, efficient optimization, and multithreading. J. Comput. Chem..

