# Supplementary material for: Combined Effects of ATRA and ASA on HeLa Cell Viability, MAPK/AKT-Related Protein Abundance, and Intracellular VEGF: An Integrated In Vitro and In Silico Study
Source: Pharmaceuticals (Basel). 2026 Oct 1;19(10):1556. doi: 10.3390/ph19101556 (PMC13708244; doi:10.3390/ph19101556)
Supplement: Supplementary file 1 [file pharmaceuticals-19-01556-s001.zip › pharmaceuticals-4527547-supplementary.pdf]

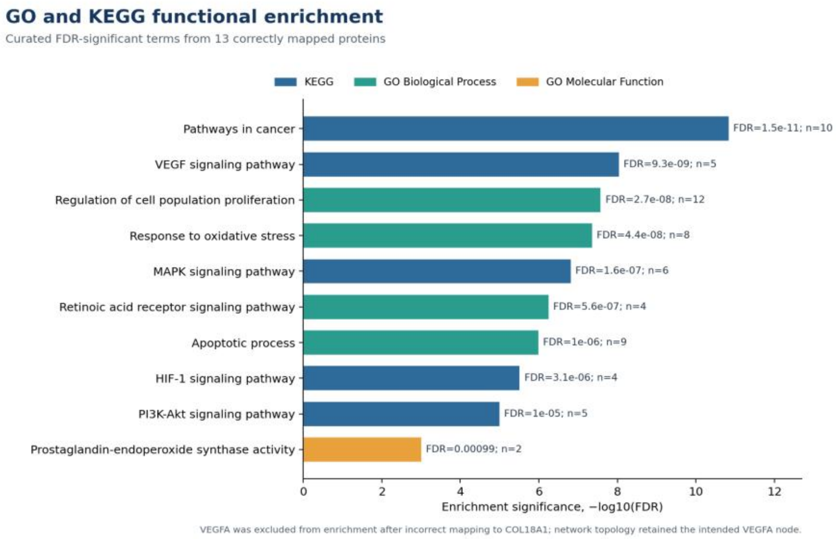

Supplementary Figure S1. GO and KEGG functional enrichment of the hypothesis-driven STRING protein set. Bars indicate enrichment significance as −log10(FDR), with labels showing the FDR value and the number of mapped genes contributing to each term. VEGFA was excluded from enrichment after incorrect mapping to COL18A1; however, the intended VEGFA node was retained in the network-topology analysis.

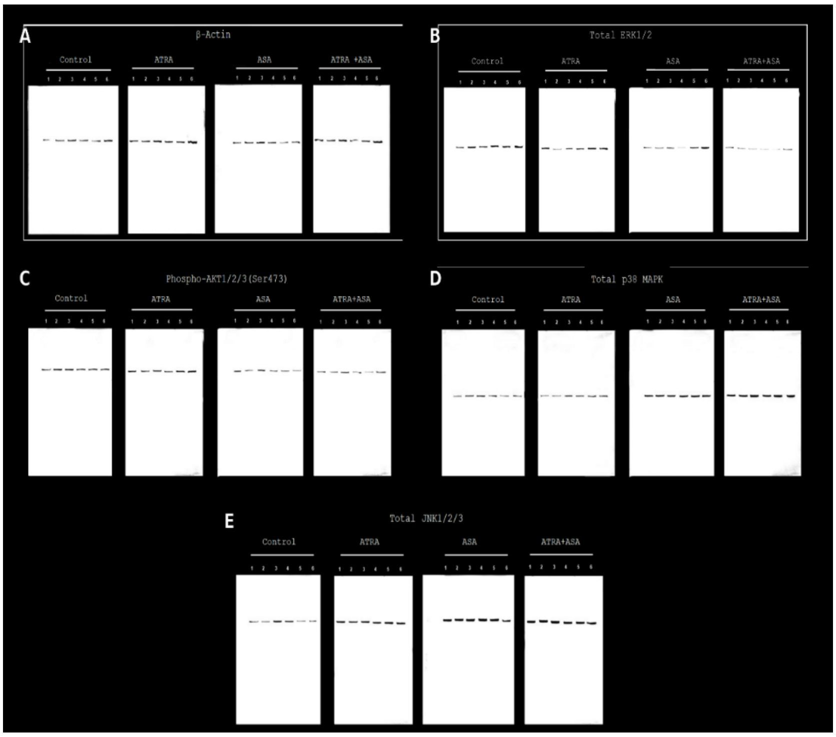

Supplementary Figure S2. Archived Western blot images used for quantitative densitometric analysis. Available archived Western blot images showing β-actin, total ERK1/2, phospho-AKT1/2/3 (Ser473), total p38 MAPK, and total JNK1/2/3 in vehicle-control, ATRA, ASA, and combined ATRA+ASA groups. Six samples are shown for each experimental group. β-Actin was used as the loading control for densitometric normalization. Following local background correction, the intensity

of each target-protein band was normalized to the corresponding β-actin band intensity from the same sample. The resulting target/β-actin ratios were expressed relative to the mean value of the vehicle-control group, which was assigned a value of 1.00. The corresponding quantitative densitometric results are presented in Figure 4. The original uncropped, full-length membrane images containing molecular-weight markers were not retained and were therefore unavailable for retrospective presentation.

Supplementary Table S1. Fixed-ratio ATRA+ASA interaction metrics. Numerical values are reported for Bliss excess inhibition, 20,000-resample bootstrap confidence intervals, P(excess > 0), Chou–Talalay combination index, and Loewe additivity index across the six tested fixed-ratio combinations.

ATRA (μM)	ASA (mM)	Bliss excess (pp)	95% bootstrap CI	CT CI	Loewe index	Overall interpretation
2.5	0.25	2.58	0.36 to 4.64	0.783	0.797	Model-dependent; Bliss additive, CT/Loewe synergy
5.0	0.50	0.37	−3.12 to 4.33	0.909	0.904	Additive
10.0	1.00	−5.64	−8.25 to −3.06	1.091	1.063	Model-dependent negative deviation
25.0	2.50	−9.74	−11.63 to −7.37	1.335	1.268	Antagonism supported
50.0	5.00	−7.14	−9.20 to −4.91	0.985	0.909	Model-dependent negative deviation
100.0	10.00	−5.74	−7.69 to −3.86	0.902	0.818	Model-dependent; Bliss negative, Loewe synergy

Note: Bliss calls used thresholds of >+5 percentage points for synergy, −5 to +5 percentage points for additivity, and <−5 percentage points for antagonism. Chou–Talalay and Loewe calls used thresholds of <0.90 for synergy, 0.90–1.10 for additivity, and >1.10 for antagonism. The combination experiment did not include its own matched control; pooled 48-h single-agent controls were used for normalization. Bootstrap intervals quantify uncertainty in Bliss deviation but do not remove this design limitation.
